# ﻿Illustrated key to the European genera of Opiinae (Hymenoptera, Braconidae), with the description of two new Palaearctic genera and two new species

**DOI:** 10.3897/zookeys.1176.104850

**Published:** 2023-08-22

**Authors:** Cornelis van Achterberg

**Affiliations:** 1 Naturalis Biodiversity Center, P.O. 9517, 2300 RA Leiden, Netherlands Naturalis Biodiversity Center Leiden Netherlands

**Keywords:** Bulgaria, *Cavopius*, Greece, Korea, new combination, new genus, new synonym, *Pseudosteres*, Turkey

## Abstract

An illustrated key to the European genera of the subfamily Opiinae (Hymenoptera, Braconidae) is presented and two new genera are described and illustrated: *Cavopius***gen. nov.** (type species: Opius (Agnopius) daghoides Zaykov & Fischer, 1983) from West and East Palaearctic regions and *Pseudosteres***gen. nov.** (type species: *Biosteresadanaensis* Fischer & Beyarslan, 2005) from West Palaearctic region. Two new species are described and illustrated: *Cephaloplitesgijswijti***sp. nov.** from Greece and *Cavopiusdepressorius***sp. nov.** from S. Korea. Opius (Hypocynodus) kilisanus Fischer & Beyarslan, 2005 is a new synonym of *Cephaloplitesmocsaryi* Szépligeti, 1897. The following new combinations are proposed: *Cavopiusdaghestanicus* (Telenga, 1950), **comb. nov.**, *C.daghoides* (Zaykov & Fischer, 1983), **comb. nov.**, *Pseudosteresadanaensis* (Fischer & Beyarslan, 2005), **comb. nov.**, *P.arenaceus* (Jakimavičius, 1986), **comb. nov.**, *P.christenseni* (Papp, 1982), **comb. nov.**, *P.pseudarenaceus* (Fischer & Beyarslan, 2005), **comb. nov.**, and *P.riphaeus* (Tobias, 1986), **comb. nov.** Keys to species are provided for *Cavopius***gen. nov.**, *Cephaloplites* Szépligeti, 1897, and *Pseudosteres***gen. nov.**

## ﻿Introduction

Opiinae is a large subfamily of the family Braconidae with ca 2,000 valid species and 39 genera according to Yu et al. (2016). It is a common group containing generally small (body length 2–5 mm) parasitoid wasps of mainly mining or fruit-infesting dipterous larvae. The subfamily has a worldwide distribution and its species have been reviewed by Fischer (1972, 1977, 1986, 1987). Wharton (e.g., 1987, 1988, 1997) published important updates and some additions for the existing keys to the genera of Opiinae, but the number of genera and the limits of some genera remain a matter of discussion, especially of *Opius* Wesmael, 1835 and of *Eurytenes* Foerster, 1863. The host of subgenera as used by Fischer (e.g., 1972) is mainly based on one character only and some specimens can be assigned to three subgenera with the key by Fischer (1972) because of intermediate conditions. In Li et al. (2013) most of the subgenera used by Fischer in his revisions were synonymised, but *Phaedrotoma* Foerster, 1863 was recognised as a valid genus for the species with symmetrical mandibles and excluded from the genus *Opius* Wesmael (following van Achterberg (1997, 2004) and van Achterberg and Salvo (1997)). However, this proved problematic because of intermediate specimens, even belonging to the same species and, therefore, *Phaedrotoma* is here synonymised with *Opius*.

Among the large collection of Opiinae in Naturalis Biodiversity Center (Leiden) two new genera were discovered and a new species of the rare genus *Cephaloplites* Szépligeti. The new taxa are described, keyed, and illustrated below and an illustrated key to the genera is provided. This paper is part of the revision of the European species of the subfamily Opiinae.

In this paper the criterium for recognition as a separate (new) genus is the possession of a set of presumably derived characters. The results of molecular research published in Li et al. (2013) support, at least partly, the choices made as far as taxa were included but also show that the inclusion of *Phaedrotoma* in *Opius* makes the latter genus more polyphyletic. Unfortunately, *Opius* s.l. lacks a set of derived characters, but we do not yet have enough knowledge of the phylogeny of the Opiinae to solve this problem.

## ﻿Materials and methods

The specimens were either collected in a Malaise trap or collected by using a sweep net. The Malaise trap specimens were chemically treated with a mixture of xylene + alcohol 96% and amylacetate, respectively (AXA-method; van Achterberg 2009). For identification of the subfamily Opiinae, see van Achterberg (1990, 1993, and 1997); for references to the Opiinae, see Yu et al. (2016).

Morphological terminology follows van Achterberg (1988, 1993), including the abbreviations for the wing venation. Measurements are taken as indicated by van Achterberg (1988): for the length and the width of a body part the maximum length and width is taken, unless otherwise indicated. The length of the mesosoma is measured from the anterior border of the mesoscutum to the apex of the propodeum and of the tergite I from the posterior border of the adductor to the medio-posterior margin of the tergite.

Observations and descriptions were made either under an Olympus SZX11 stereomicroscope. Photographic images were taken with a Canon 5Ds 50.6-megapixel camera combined with a Canon MP-E 65 mm f/2.8 1–5× Macro lens, Laowa Macro Twin flash KX-800 and an electronic WeMacro Z-stepper rail. The photos were stacked with Helicon Focus 7 software. The type specimens are deposited in the Naturalis collection (**RMNH**) at Leiden. **NMW** and **ZISP** stand for Naturhistorisches Museum at Vienna and Zoological Institute at St. Petersburg, respectively.

### ﻿Key to European genera of Opiinae

**Table:** 

1	Vein 2-SR of fore wing absent (a); first subdiscal cell of fore wing open apically (b); vein cu-a of hind wing absent (c); segments of maxillary palp usually shorter (d); [clypeus wide, short, and impressed; hind wing narrow]	**2**
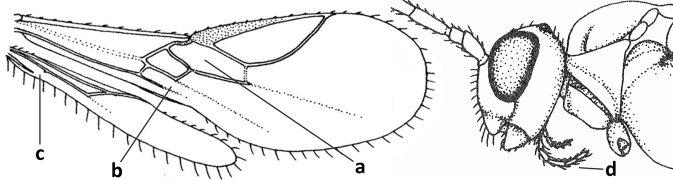		
–	Vein 2-SR of fore wing present (aa), rarely absent; first subdiscal cell of fore wing at least partly closed by vein 3-CU1 apically (bb); vein cu-a of hind wing nearly always present (cc); segments of maxillary palp often elongate (dd)	**3**
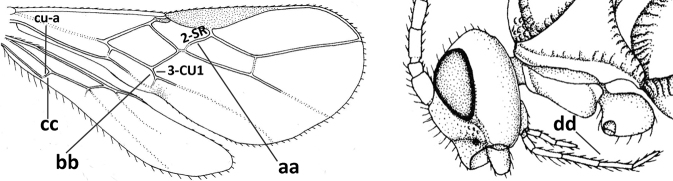		
2	Marginal cell of fore wing open apically (a) and long; veins m-cu (b) and r-m (c) of fore wing absent; occipital carina absent laterally (d); [metasomal tergites I and III more or less coriaceous or rugulose; tergites IV–VI largely retracted]	***Indiopius* Fischer, 1966**
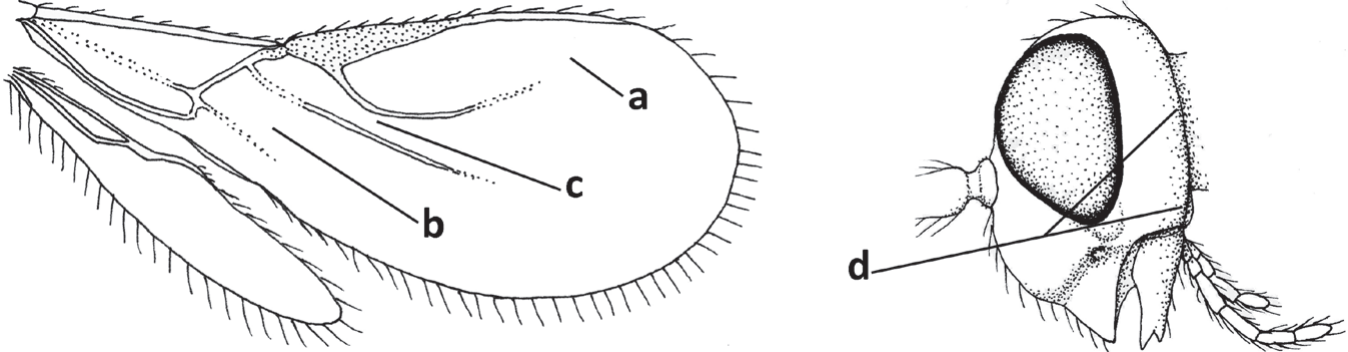		
–	Marginal cell of fore wing closed apically (aa) and shortened; veins m-cu (bb) and r-m (cc) of fore wing present; occipital carina present laterally (dd)	***Pokomandya* Fischer, 1959**
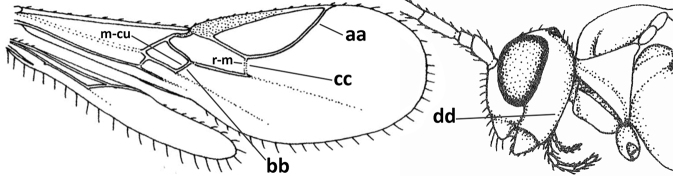		
3	Medio-laterally occipital carina near level of middle of eye strongly curved, resulting in an oblique part of carina or crest (a); vein 1-R1 of fore wing shorter than pterostigma (b); clypeus flat and short (c)	***Hoplocrotaphus* Telenga, 1950**
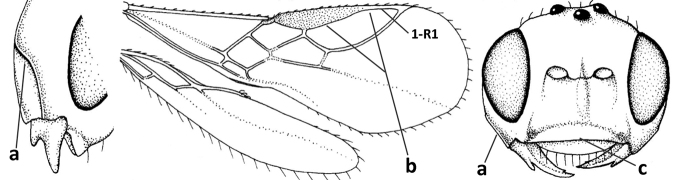		
–	Medio-laterally occipital carina straight or slightly curved (aa) or carina absent; vein 1-R1 of fore wing usually as long as or longer than pterostigma (bb); if shorter then clypeus more or less convex and longer (cc)	**4**
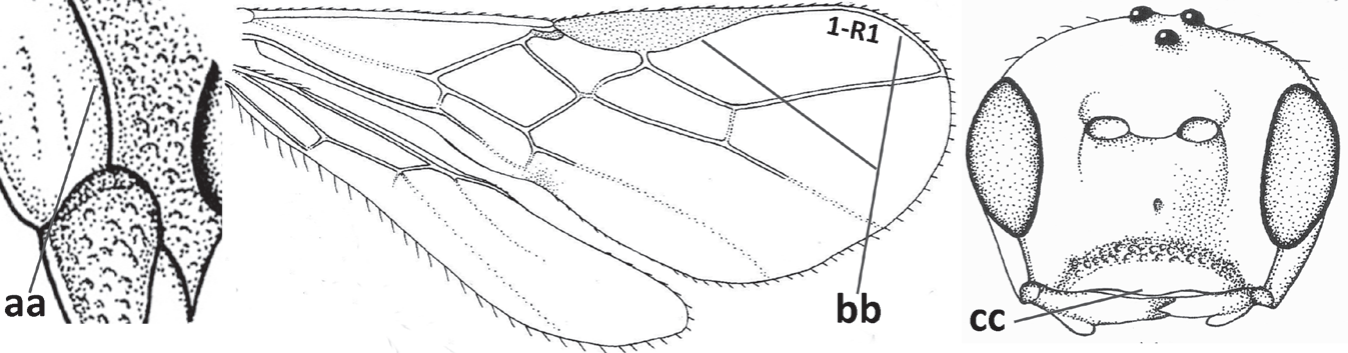		
4	Occipital carina completely absent (a) **and** vein CU1b of fore wing absent (b); convex labrum with long curved setae forming a comb ventrally (observe in oblique view and mandibles at least partly open: c); [body black; malar suture slightly longer than basal width of mandible; anterior tentorial pits close to apical margin of clypeus; ovipositor sheath shorter than apical height of metasoma]	***Desmiostoma* Foerster, 1863**
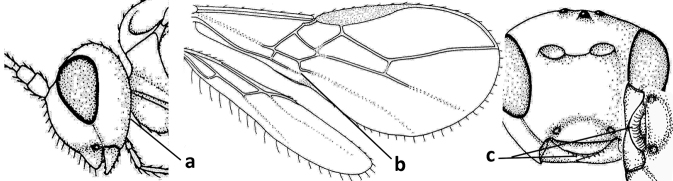		
–	Occipital carina present laterally (aa); if rarely absent then vein CU1b of fore wing present (bb); labrum flattened and without comb of setae ventrally (cc)	**5**
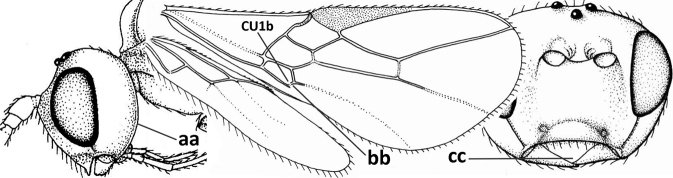		
5	Inner side of hind tibia with 1 carina or 3 or 4 carinulae baso-laterally in a glabrous area (a); pronope slit-like, round or elliptical, often large (occupying most of medio-dorsal part of pronotum) and deep (b); vein 1-M of fore wing more or less curved (c); [propodeum often coarsely (reticulate-)rugose and with more or less curved transverse carina dorso-laterally; dorsope absent or slightly impressed because of strongly developed dorsal carinae; hypostomal flange usually large below base of mandible]	**6**
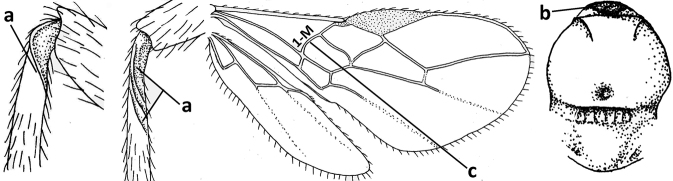		
–	Inner side of hind tibia without carina or carinulae baso-laterally and largely setose (aa); pronope usually round and smaller or pronotum only with shallow transverse groove (bb); vein 1-M of fore wing usually straight (cc)	**7**
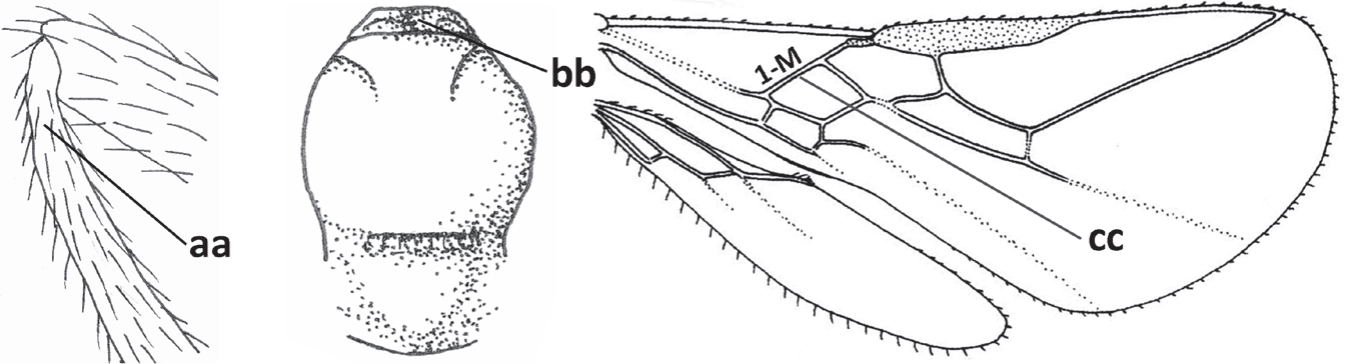		
6	Inner side of hind tibia with 3 or 4 fine carinulae (a; rarely 5 or 6 carinulae); mandible distinctly asymmetrical, more narrowed submedially (b) and widened ventro-basally, but less so in *O.pactus*)	***Opiognathus* Fischer, 1972**
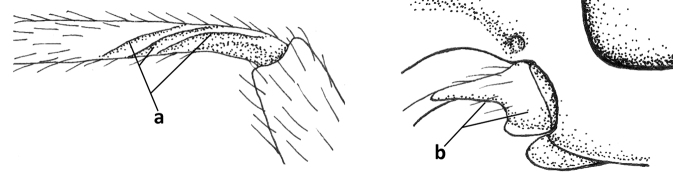		
–	Inner side of hind tibia with one rather lamelliform carina (aa); mandible nearly symmetrical, gradually narrowed submedially (bb) and usually slightly widened ventro-basally	***Utetes* Foerster, 1863 s.str.**
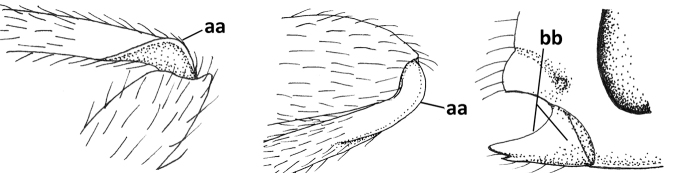		
7	Occipital carina above mandibular base curved and (just) meeting hypostomal carina (a); medio-posterior depression of mesoscutum present (b), only rarely absent or obsolescent; dorsope absent (c); malar space comparatively long (d); [if dorsope present and medio-posterior depression of mesoscutum absent, see *Atormus* van Achterberg]	***Apodesmia* Foerster, 1863**
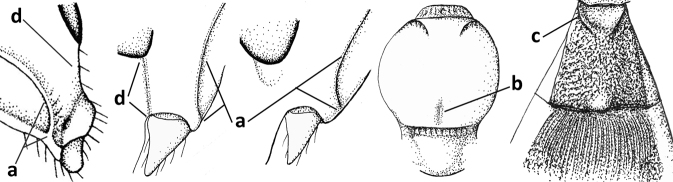		
–	Occipital carina not or slightly curved ventrally and remain removed from hypostomal carina (aa) or occipital carina absent laterally (aaa); medio-posterior depression of mesoscutum absent (bb) or present (bbb); dorsope present (cc) or absent (ccc); if absent then malar space usually short (dd)	**8**
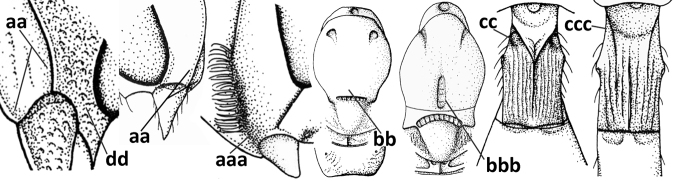		
8	Crenulate depression above eye present (a); prepectal carina more or less developed ventrally (b); vein SR1 of fore wing reduced apically, resulting in an open marginal cell (c); hind tibia and tarsus, and tarsal claws very slender (d); [medio-posteriorly scutellum with continuation of lateral elevated area; malar suture absent or obsolescent]	***Ademon* Haliday, 1833**
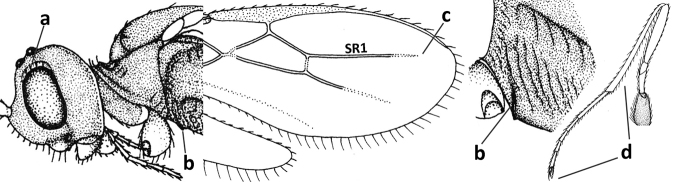		
–	Crenulate depression above eye absent (aa); prepectal carina absent ventrally (bb); vein SR1 of fore wing completely sclerotised, reaching margin of wing and resulting in a closed marginal cell (cc); hind tibia and tarsus, and tarsal claws medium-sized (dd)	**9**
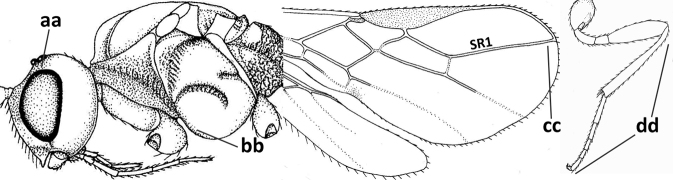		
9	Face with pair of tubercles below antennal sockets (a); epistomal suture with pair of large oblique and long pubescent depressions below facial tubercles (b); scape strongly compressed (c); scutellar sulcus narrow (d); [mandible with wide basal tooth]	***Cephaloplites* Szépligeti, 1897**
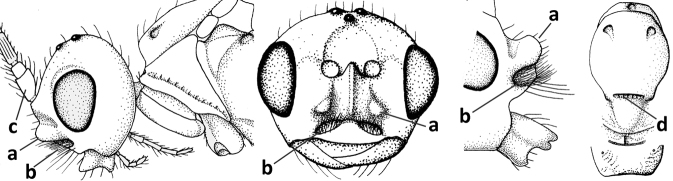		
–	Face without tubercles (aa); epistomal suture without large depressions (bb); scape at most weakly compressed (cc); scutellar sulcus usually wider (dd)	**10**
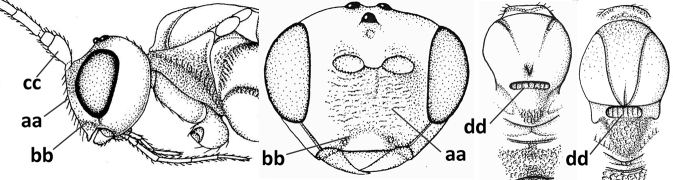		
10	Metasomal tergite II distinctly (1.3–1.9×) longer than tergite III and differentiated (a); tergite II bordered posteriorly by a curved second metasomal suture (b); vein 3-SR of fore wing approx. as long as vein 2-SR or slightly longer (c); tergite II longitudinally striate or rugose (d)	**11**
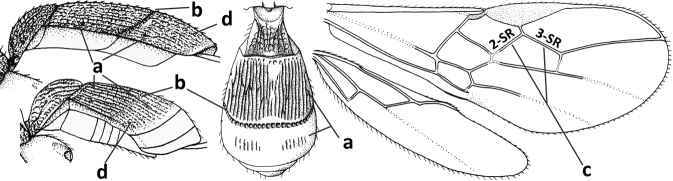		
–	Tergite II 0.7–1.1× as long as tergite III (aa), but frequently tergite II hardly or not differentiated; second metasomal suture nearly straight (bb) or absent (bbb); **if** metasoma shortened then vein 3-SR of fore wing distinctly longer than vein 2-SR (cc; *Opiusagnesae*) and tergite II smooth or granulate (dd); [if metasoma shortened and propodeum with transverse carina subbasally, see *Coleopioides* van Achterberg & Li]	**13**
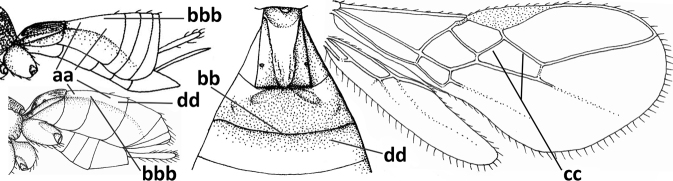		
11	Around base of middle coxa with a circular carina (a); tergite III without sharp lateral crease (b); occipital carina present latero-dorsally and rather protruding in lateral view (c); head less elongate in anterior view (d); [dorsal carinae of tergite I united basally and medially forming a median carina; mesoscutum smooth laterally]	***Bitomoides* van Achterberg, 2004**
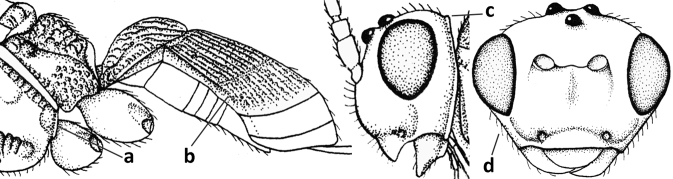		
–	Around base of middle coxa without a circular carina (aa); tergite III with a sharp lateral crease (bb); occipital carina absent latero-dorsally or if present usually less protruding in lateral view (cc); head more elongate in anterior view (dd); [mesoscutum finely crenulate laterally; dorsal carinae of tergite I usually separated basally and medially without a median carina]	**12**
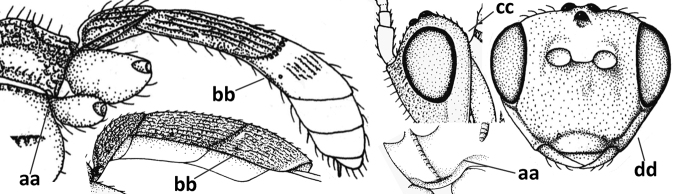		
12	Medio-posterior depression of mesoscutum present (a); malar suture complete and distinctly impressed (b); posterior 1/2 of notauli entirely or largely absent (c)	***Orientopius* Fischer, 1966**
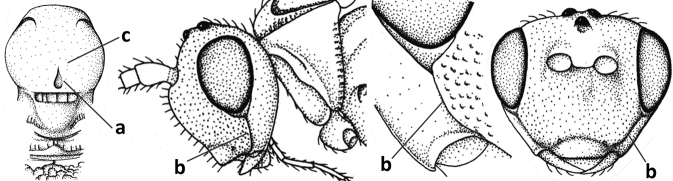		
–	Medio-posterior depression of mesoscutum absent (aa); malar suture absent or obsolescent (bb); posterior 1/2 of notauli complete (cc); [laterope distinct]	***Coleopius* Fischer, 1964**
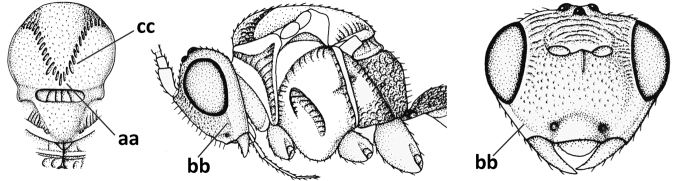		
13	Vein r of fore wing approx. as long as vein 2-SR (a), slightly curved, not angled with vein 3-SR and issued near base of pterostigma (b); basal third of pterostigma much narrower than apical third (c); dorsal carinae of metasomal tergite I largely obsolescent (d); [dorsope often minute or obsolescent; spiracles of tergite I situated near middle of notum; malar suture distinct]	***Eurytenes* Foerster, 1863 s.str.**
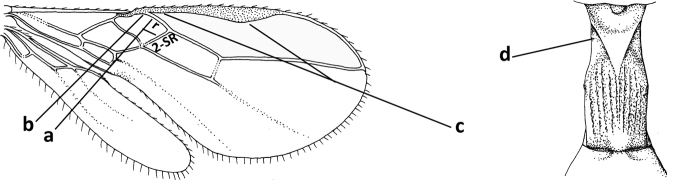		
–	Vein r of fore wing much shorter than vein 2-SR (aa), straight, more or less angled with vein 3-SR and issued distinctly removed from base of pterostigma (bb); basal 1/3 of pterostigma approx. as wide as apical 1/3 or slightly narrower (cc); dorsal carinae of tergite I usually distinctly developed (dd)	**14**
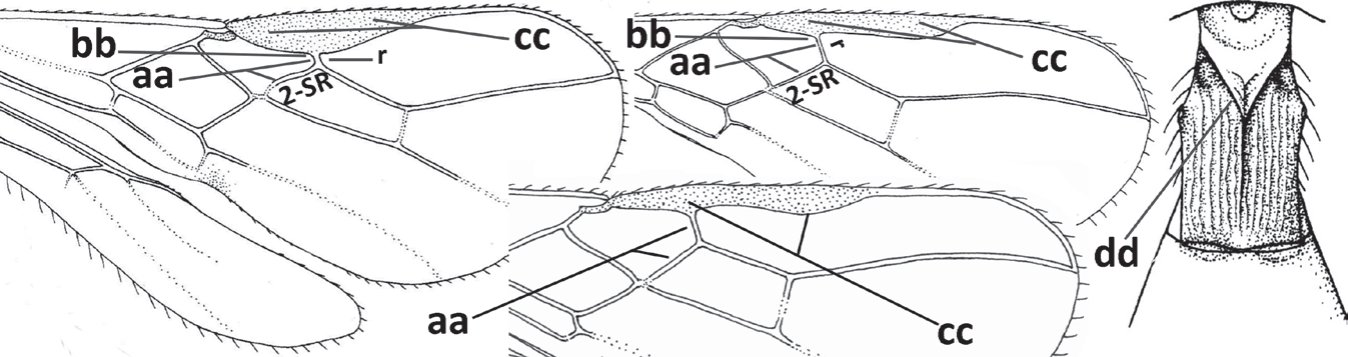		
14	Dorsope present (a); if with small dorsope then mandible with a short lamella ventro-basally (b) or pterostigma slightly widened subapically (c); vein m-cu of hind wing usually present, at least as a partly pigmented trace (d); [medio-posterior depression of mesoscutum present, rarely absent or obsolescent; antenna often longer than 1.3× length of fore wing]	**15**
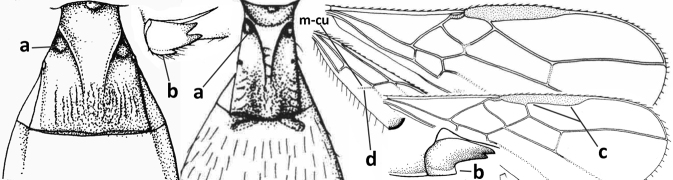		
–	Dorsope absent (aa); mandible usually without a short lamella ventro-basally or with a narrow carina along most of mandible ventrally (bb); pterostigma narrowed subapically (cc); vein m-cu of hind wing often absent (dd), but present in *Diachasma* and *Bathystomus*; [antenna usually less than 1.3× length of fore wing]	**21**
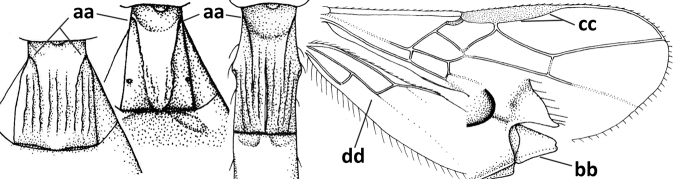		
15	Mandible not or slightly twisted medially and its second tooth distinctly visible in lateral view (a), wide apically and its outer side convex (b); vein 3-SR of fore wing 1.3× as long as vein 2-SR or less (b), but 1.2–1.6× in *Pseudosteres*	**16**
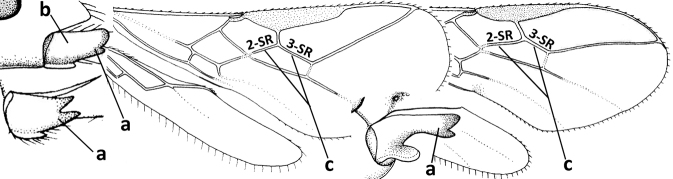		
–	Mandible more or less twisted medially and its second tooth hardly or not visible in lateral view (aa), triangular and its outer side often flattened or weakly convex (bb), but convex in *Xynobius* and *Atormus*; vein 3-SR of fore wing 1.4–2.5× longer than vein 2-SR (cc)	**18**
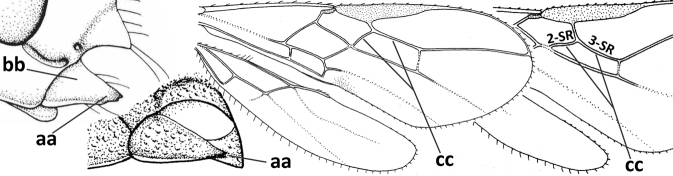		
16	Mandibles asymmetrical, with a large ventro-basal tooth or lobe (a); hypoclypeal depression medium-sized to narrow (b), rarely absent; pterostigma wide elliptical or elongate triangular (c); pronope absent or nearly so (d); [vein 3-SR of fore wing 1.2–1.6× longer than vein 2-SR; vein m-cu of fore wing sometimes curved]	***Pseudosteres* gen. nov.**
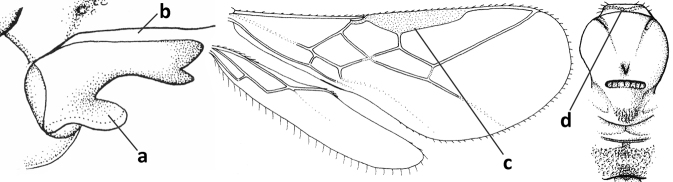		
–	Mandible symmetrical, without ventro-basal tooth, at most with an acute basal lamella (aa); hypoclypeal depression absent (bb) or narrow; shape of pterostigma variable (cc), **if** comparatively wide elliptical or triangular (ccc) then usually pronope large (dd); [vein m-cu of fore wing straight]	**17**
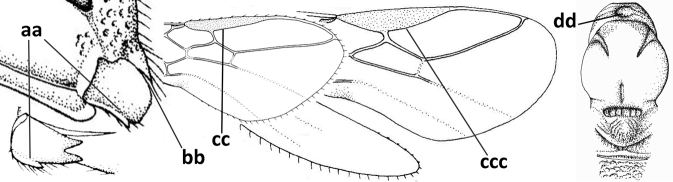		
17	Mesopleuron narrowed ventrally (a); epistomal suture absent (b); basal 1/2 of vein M+CU1 of fore wing entirely or largely sclerotised (c); clypeus with dense long setae, and convex (d); [mandible without distinct ventro-basal carina, but sometimes weakly developed]	***Chilotrichia* Foerster, 1863**
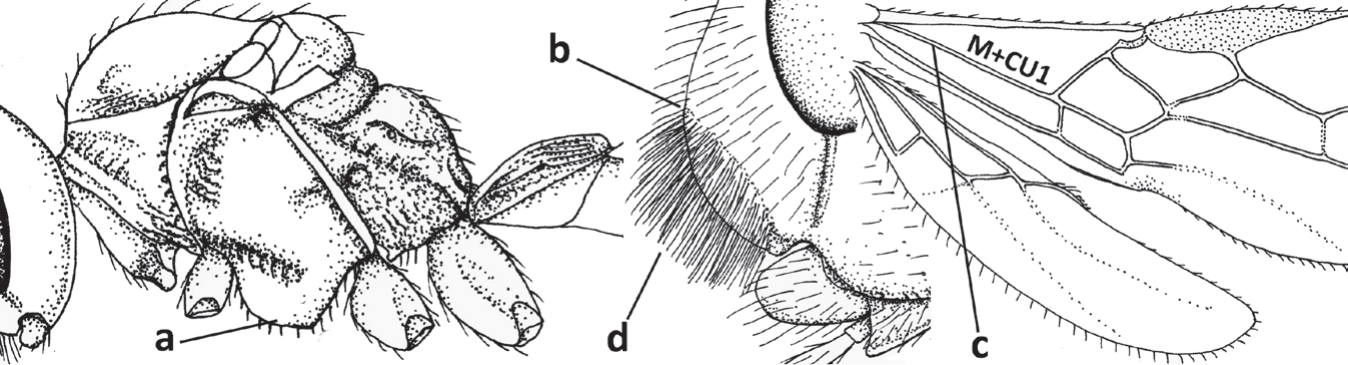		
–	Mesopleuron wider and rounded ventrally (aa); epistomal suture present (bb); if shallow then basal 1/2 of vein M+CU1 of fore wing largely unsclerotised (cc); clypeus with less dense and shorter setae, if setae longer and denser then clypeus flattened (dd)	***Biosteres* Foerster, 1863**
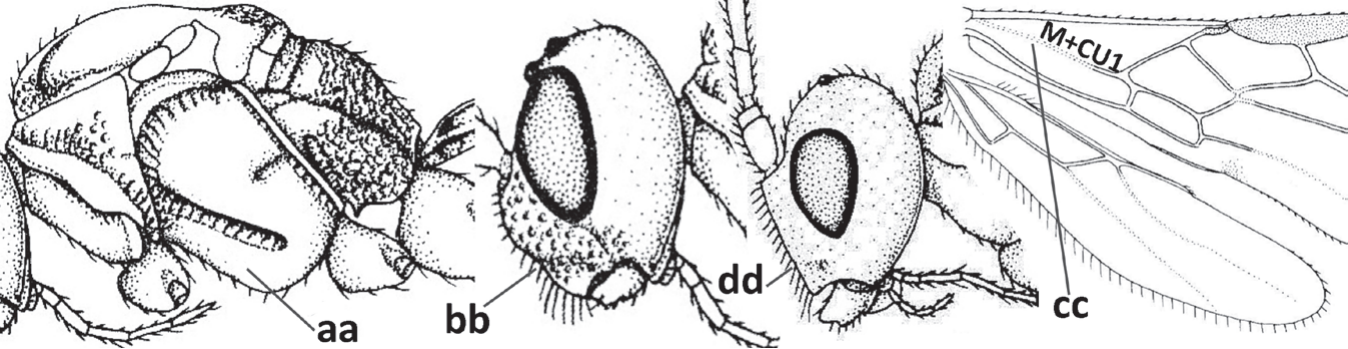		
18	Hypoclypeal depression present, large, and medially ventral margin of clypeus near upper level of condyles of mandibles (“subcyclostome condition”; a); mandible symmetrical basally (b) and outer side convex; [malar suture deep]	**19**
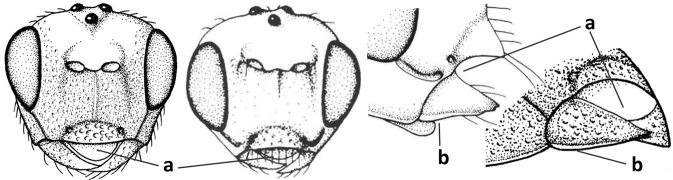		
–	Hypoclypeal depression absent or narrow, and medially ventral margin of clypeus more or less below upper level of condyles of mandibles (“mouth closed” or nearly so; aa); mandible often widened ventro-basally (asymmetrical: bb) and outer side usually flattened or weakly convex	**20**
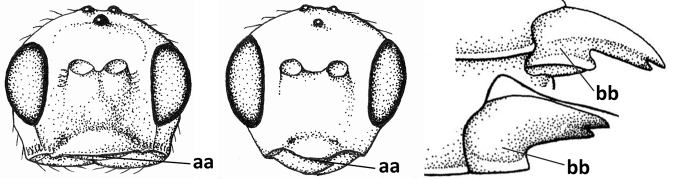		
19	Propleuron without fine oblique carina subapically (a); medio-posterior depression of mesoscutum present (b); first subdiscal cell of fore wing subparallel-sided (c)	***Xynobius* Foerster, 1863**
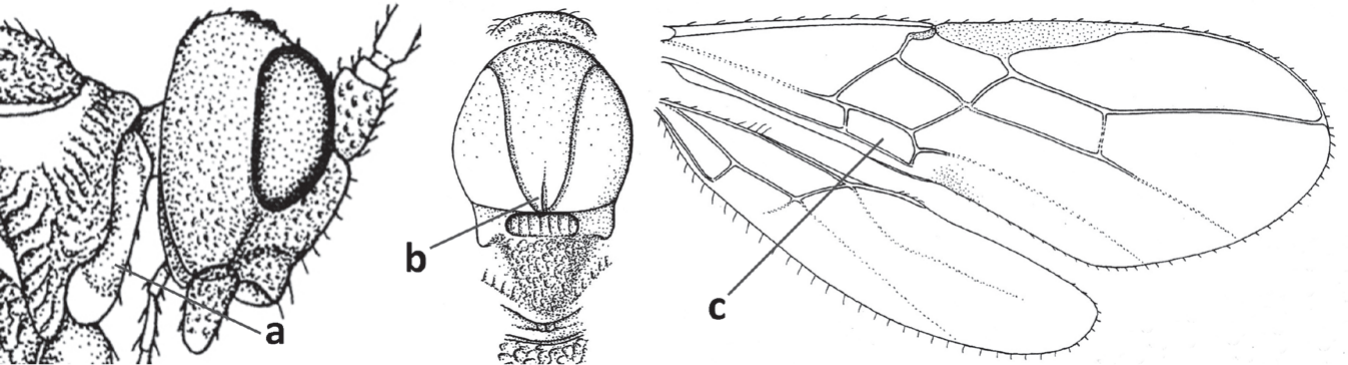		
–	Propleuron with (oblique or transverse) carina subapically (aa); medio-posterior depression of mesoscutum absent (bb); first subdiscal cell of fore wing widened distally (cc); [metasomal tergite III evenly setose, but sometimes sparsely so; hypostomal flange protruding ventrally]	***Atormus* van Achterberg, 1997**
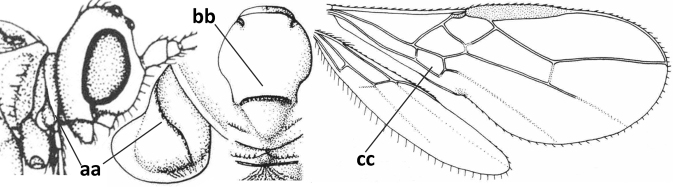		
20	Scutellum elevated above level of mesoscutum (a); below precoxal sulcus with a second finely sculptured sulcus (= sternaulus: b), but sometimes only medially superficially impressed; vein 1-M of fore wing curved (c) and vein r short (d); malar space crenulate ventrally (e); [vein m-cu of fore wing strongly converging posteriorly to vein 1-M or subparallel]	***Biophthora* Foerster, 1863**
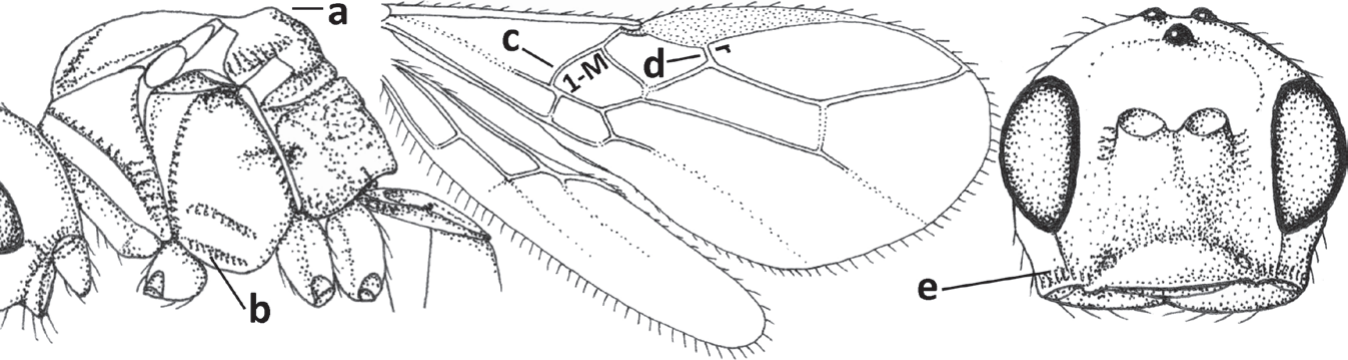		
–	Scutellum hardly or not elevated above mesoscutum (aa); sternaulus absent (bb), at most with one groove (= precoxal sulcus) present; vein 1-M of fore wing straight (cc) or nearly so and vein r medium-sized to long (dd); malar space smooth ventrally (ee)	***Opiostomus* Fischer, 1972**
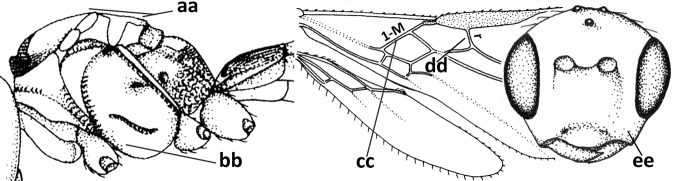		
21	Vein 3-SR of fore wing 0.8–1.3× vein 2-SR (a); if rarely 1.3–1.5× then basal 1/2 of mandible symmetrical (b); i.e., without a ventral carina or tooth) and vein m-cu of hind wing present (at least as a distinct partly pigmented trace: c); length of fore wing usually more than 3 mm	**22**
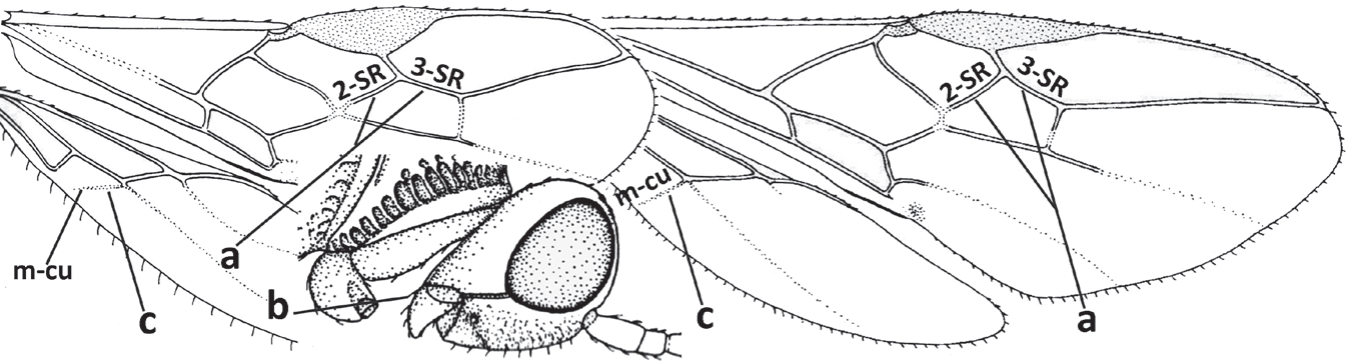		
–	Vein 3-SR of fore wing 1.3× vein 2-SR or longer (aa); if 1.0–1.5× then basal 1/2 of mandible with a narrow ventral carina (bb) or baso-ventrally widened (asymmetrical: bbb) and/or vein m-cu of hind wing absent (cc) or obsolescent; length of fore wing usually less than 3 mm	**25**
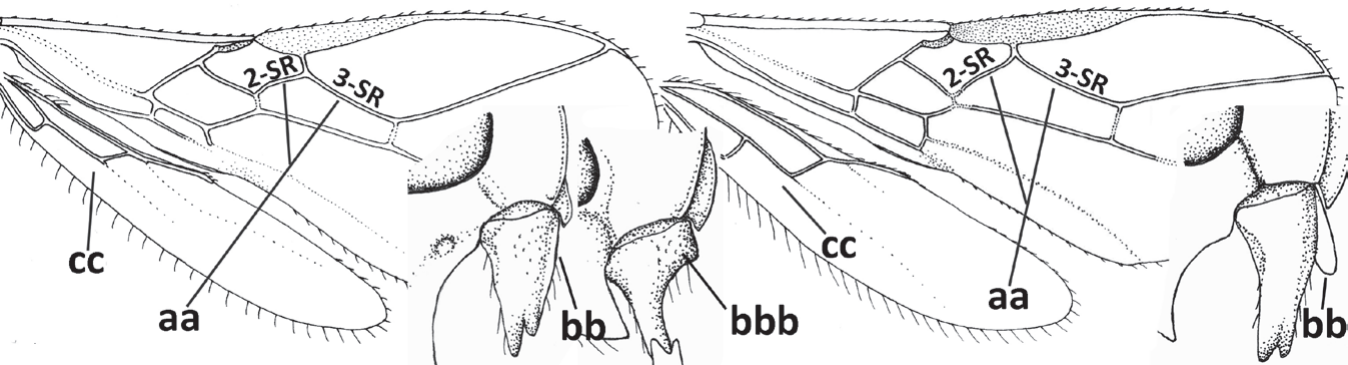		
22	Ventral margin of clypeus roundly protruding and without distinct hypoclypeal depression, at most with a narrow slit (a); fore wing pointed apically (b); ovipositor sheath long (c), usually as long as fore wing or longer; parasitoids of Tephritidae	***Diachasmimorpha* Viereck, 1913**
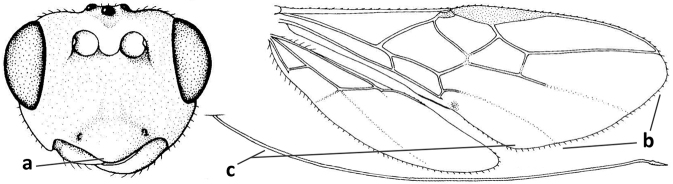		
–	Ventral margin of clypeus almost straight or slightly concave, and with a distinct hypoclypeal depression (aa); fore wing broadly rounded apically (bb); ovipositor sheath short (cc), approx. as long as apical height of metasoma or concealed; parasitoids of other dipterous hosts	**23**
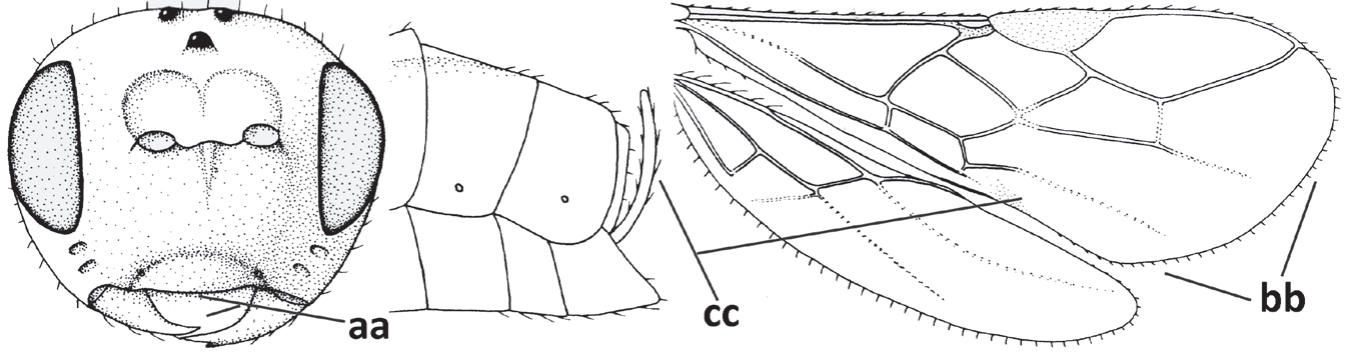		
23	Precoxal sulcus absent or largely so (a); metasomal tergite I 2.0–2.5× longer than its apical width (b); metanotum with median carina posteriorly (c); malar suture complete and oblique (d); [metasomal tergites II and III of ♀ distinctly compressed; tergite II smooth]	***Bathystomus* Foerster, 1863**
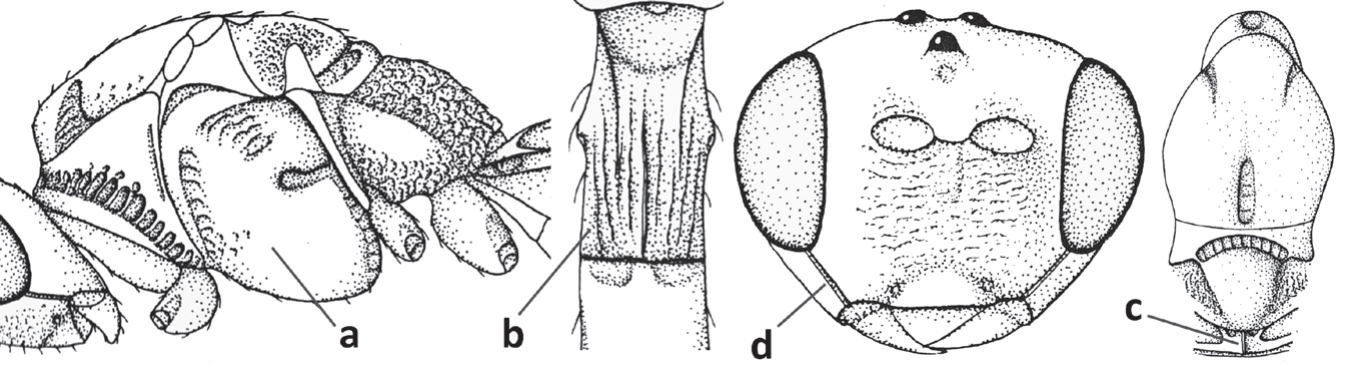		
–	Precoxal sulcus present and often wide on anterior 1/2 of mesopleuron (aa); length of tergite I 1.0–1.5× its apical width (bb); metanotum without median carina posteriorly or weakly developed (cc); malar suture absent or obsolescent (dd); [tergites II and III of ♀ not compressed]	**24**
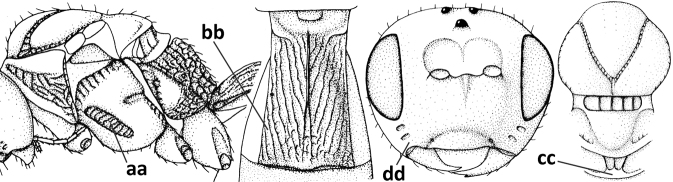		
24	Clypeus concave medio-ventrally (a); tergite II of ♀ with coarse striation (b); mandible strongly twisted basally (c); antennal segments slender (d); parasitoids of Psilidae; [mesosoma ~ 1.8× longer than high in lateral view; metanotum finely crenulate posteriorly; eyes diverging ventrally]	***Atoreuteus* Foerster, 1863**
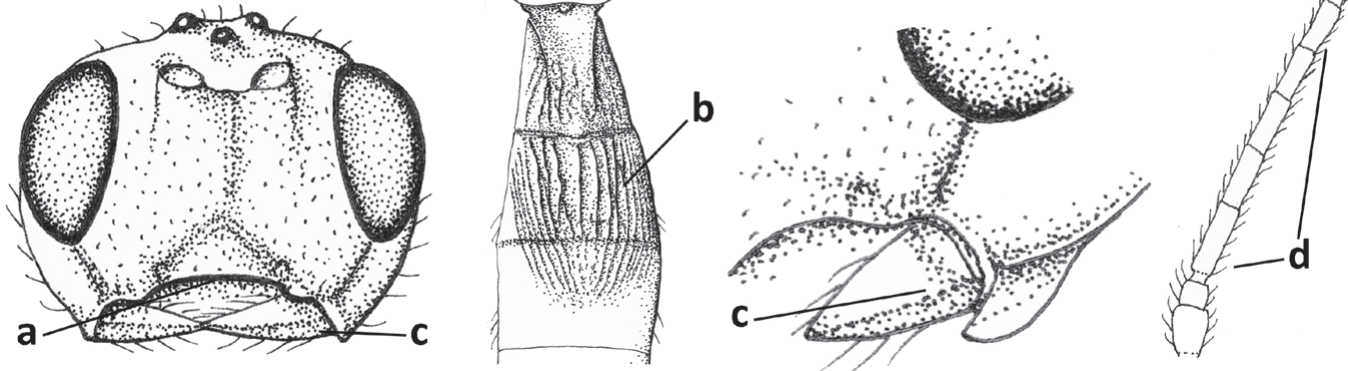		
–	Clypeus straight or protruding medio-ventrally (aa); tergite II of ♀ smooth or with very fine sculpture (bb); mandible not twisted basally (cc); antennal segments robust (dd); parasitoids of Anthomyiidae	***Diachasma* Foerster, 1863**
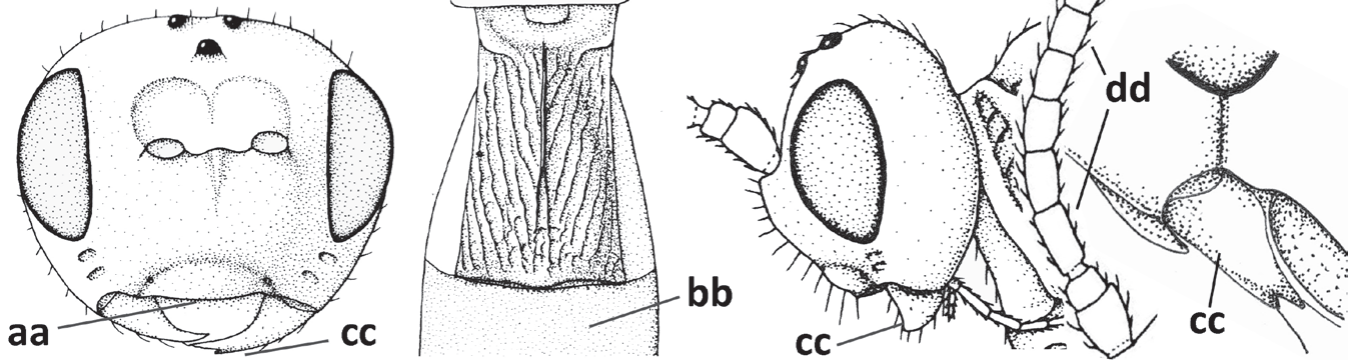		
25	Ventral 1/2 of occiput with conspicuous and more or less curved setae (a); occipital carina widely absent ventrally (b); hypopygium of ♀ acute apically, nearly as long as apical height of metasoma or somewhat shorter (c) and propodeum largely smooth (d)	***Cavopius* gen. nov.**
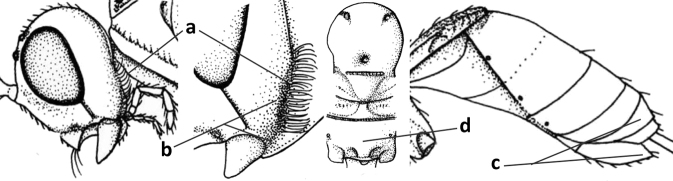		
–	Ventral 1/2 of occiput glabrous (aa); occipital carina present (bb), at most narrowly absent ventrally; hypopygium of ♀ usually obtuse apically (cc), if acute then acute part longer than basal part (ccc) and/or anteriorly propodeum with medio-longitudinal carina (dd) and/or partly sculptured	**26**
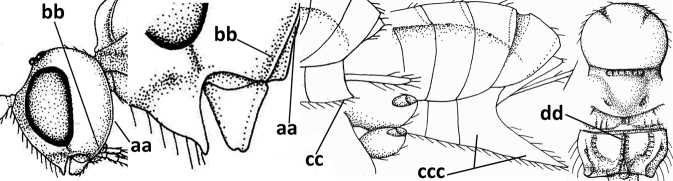		
26	Scutellum distinctly protruding above level of mesoscutum (a); hind femur very robust, 2–3× longer than wide (b); labrum slanted backwards, leaving a large space below clypeus (c); medio-anterior veins of hind wing of ♂ strongly widened (d); [hypopygium of ♀ 0.3–0.5× as long as metasoma and apically usually more or less protruding; dorsal face of propodeum strongly transverse; hind wing narrow]	***Psyttoma* van Achterberg & Li, 2012**
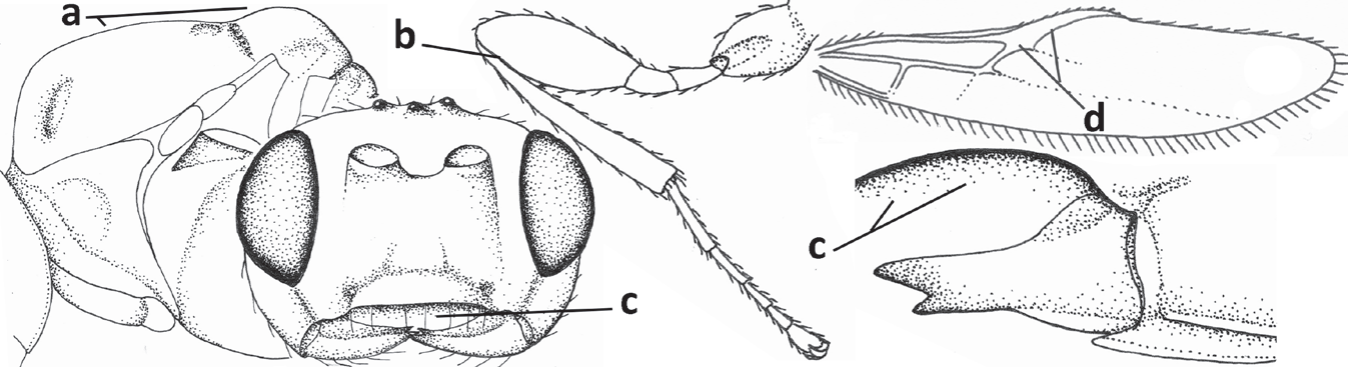		
–	Scutellum at level of mesoscutum (aa); hind femur more slender, 3–5× longer than wide (bb); labrum normal, without large space below clypeus (cc); medio-anterior veins of hind wing of ♂ narrow (dd)	**27**
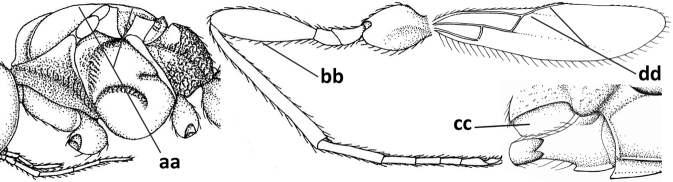		
27	Hypopygium of ♀ more narrowed apically (a), acute, 0.3–0.5× as long as metasoma (b); pronope absent or small in dorsal view (c); lateral carina of mesoscutum absent in front of tegula (d); vein 1-M of fore wing straight or nearly so (e); propodeum often with medium-sized medio-longitudinal carina connected to narrow triangular areola (f), but largely obscured by sculpture in *P.cyclogaster*; [tergite II (if differentiated) distinctly shorter than tergite III or of similar length; hind wing and hypoclypeal depression wide; antenna of ♀ 1.3–1.6× as long as fore wing]	***Psyttalia* Walker, 1860**
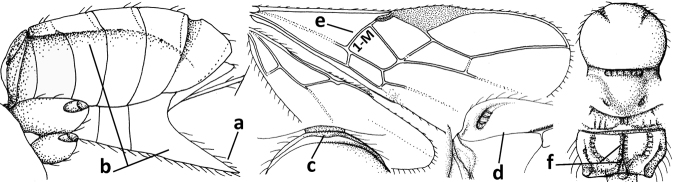		
–	Hypopygium of ♀ less narrowed apically (aa) and usually shorter than 0.3× length of metasoma (bb); pronope usually large to medium-sized (cc); **if** absent then in front of tegula with fine lateral carina of tegula (dd; sometimes area aciculate) **or** vein 1-M of fore wing curved (ee); medio-longitudinal carina of propodeum usually absent, **if** present then short and either no or wide areola (ff), rarely carina hardly developed (fff); [metasomal tergite II approx. as long as tergite III]	**28**
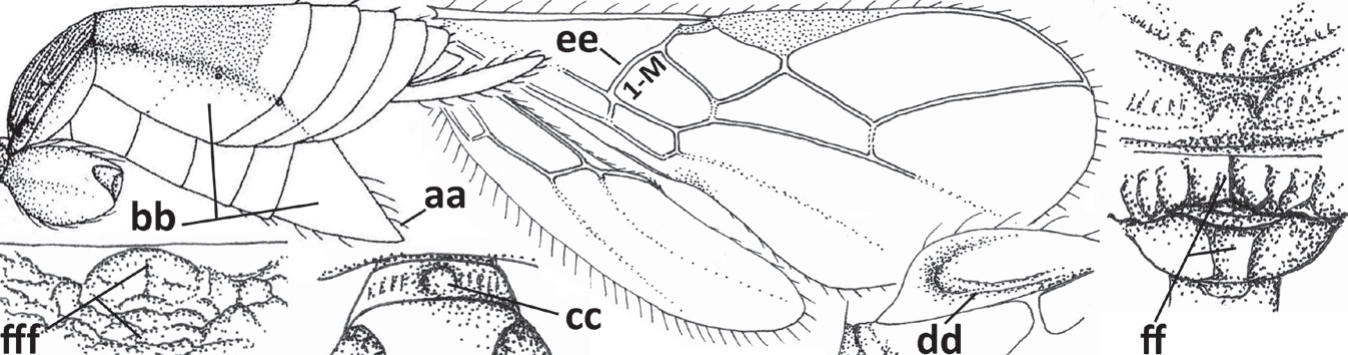		
28	Medio-posterior triangular area of mesosternum coarsely reticulate (a); occipital carina complete medio-dorsally or nearly so (b) and distinctly lamelliform dorso-laterally (c); frons distinctly granulate (d)	***Neopius* Gahan, 1917**
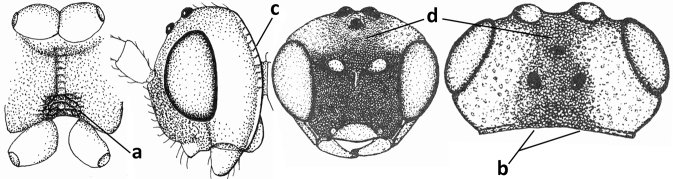		
–	Mesosternum largely smooth medio-posteriorly (aa); occipital carina widely interrupted medio-dorsally (bb); **if** more or less complete dorsally then occipital carina narrow lateral (cc) and/or frons smooth (dd)	**29**
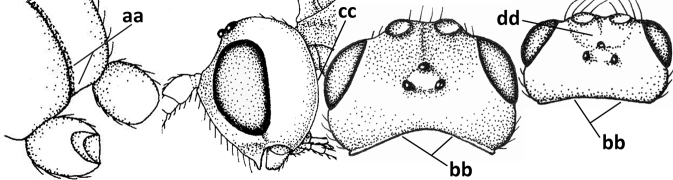		
29	Face conspicuously long and densely setose medio-dorsally (a); vein 1A+2A & 1-1A of fore wing distinctly bent and reaching posterior border of wing (b); thick ventral margin of clypeus distinctly concave medio-apically (c; medially distinctly above upper level of mandibular condyles); occipital carina fine and remaining far removed from hypostomal carina ventrally (d); [metasoma (except dark brown tergite I) orange-yellow]	***Eutrichopsis* Foerster, 1863**
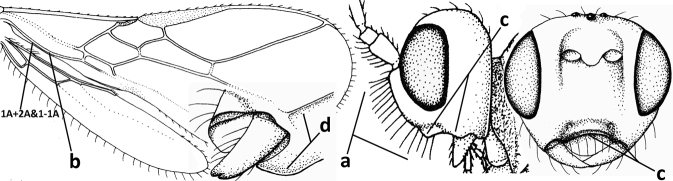		
–	Face without conspicuous setosity (aa); vein 1A+2A & 1-1A of fore wing straight or nearly so and remaining removed from posterior border of wing (bb); ventral margin of clypeus below upper level of mandibular condyles (cc) or approx. same level (ccc)) and usually thin; occipital carina usually more developed and closer to hypostomal carina ventrally (dd)	**30**
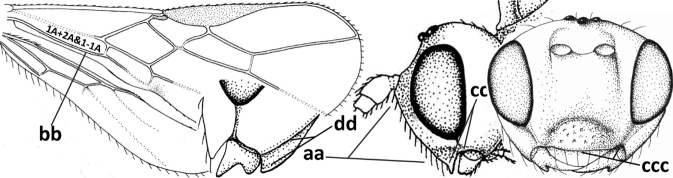		
30	Medio-longitudinal carina of propodeum present anteriorly (a), if very short and reduced or obscured by coarse reticulation, then with transverse elements of rugosity subanteriorly (aaa); vein m-cu of fore wing gradually merging into 2-CU1 and sublinear with vein 2-M (b); mandible symmetrical or nearly so (c); vein CU1b of fore wing distinct and medium-sized to small (d); [hypoclypeal depression medium-sized to large; medio-posterior depression of mesoscutum absent and precoxal sulcus distinctly crenulate]	***Rhogadopsis* Brèthes, 1913**
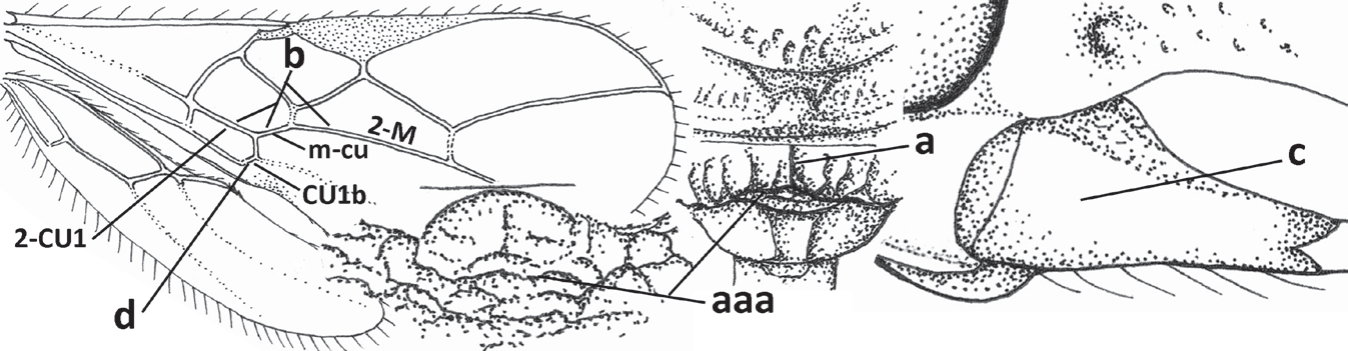		
–	Medio-longitudinal carina of propodeum absent and area smooth (aa), **if** rugose then without distinct transverse elements; vein m-cu of fore wing angled with veins 2-CU1 and vein 2-M (bb); shape of mandible varies from more or less asymmetrical and strongly narrowed medially (cc) to symmetrical and slightly narrowed medially (ccc); vein CU1b of fore wing short or absent (dd)	***Opius* Wesmael, 1835 s.l.**
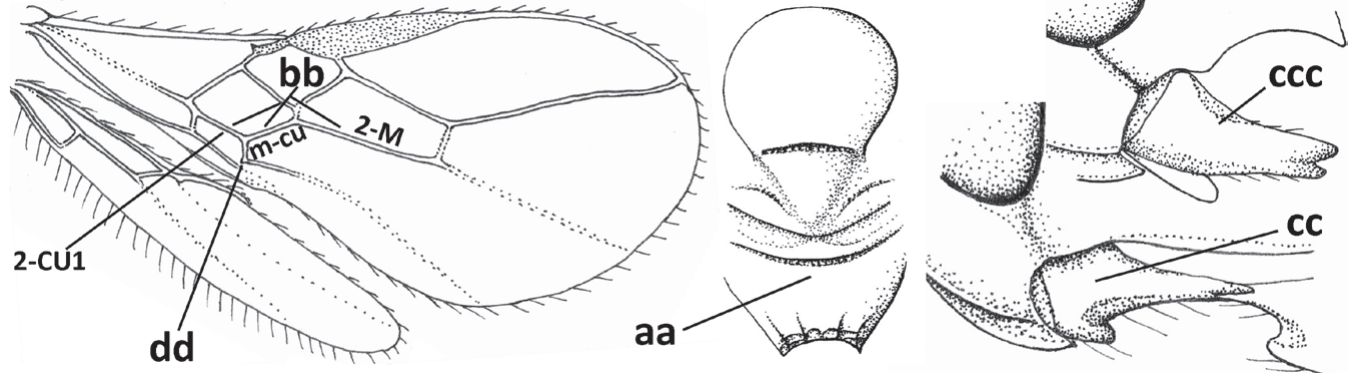		

## ﻿Taxonomy

### ﻿ Cavopius gen. nov.

Taxon classificationAnimaliaHymenopteraBraconidae

3D82A176-01E4-5556-B55A-5C5D308941BA

https://zoobank.org/D9D463B6-14A6-4338-9E8F-20DEDD152578

Figs 1 , 2–10 , 11–23 , 24–33

#### Type species.

Opius (Agnopius) daghoides Zaykov & Fischer, 1983.

#### Etymology.

From *cavus* (Latin for hollow) and the generic name *Opius* Wesmael, because of the long and curved setae make a kind of cave at the back of the head (Fig. 13). Gender: masculine.

#### Diagnosis.

**Figure 1.**
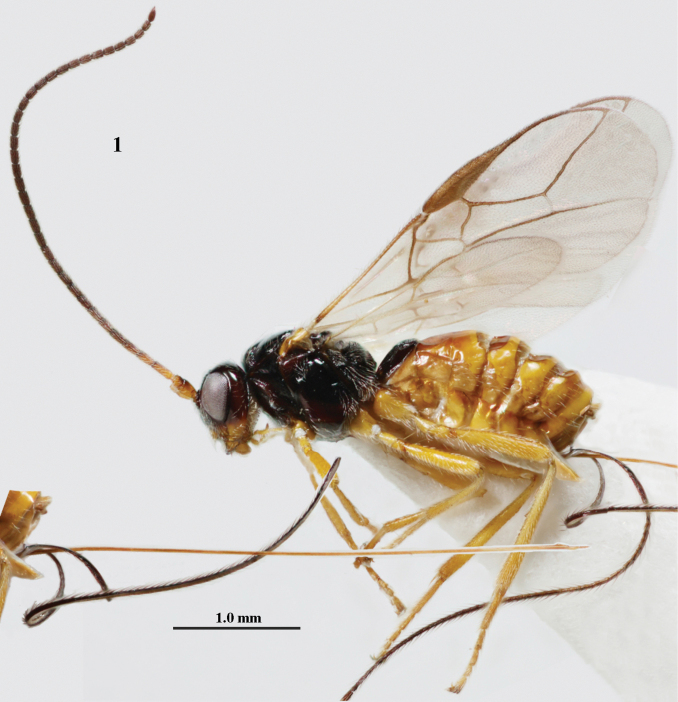
*Cavopiusdepressorius* sp. nov., holotype, ♀, S. Korea (Hudong-Li), habitus lateral.

Antenna with 26–37 segments and 1.1–1.2× as long as fore wing (latter unknown of *C.daghestanicus*); ventral half of occiput with medium-sized to large area of long conspicuous and usually curved setae (Figs 13, 17, 25, 31); occipital carina absent ventrally (from lower level of eye downwards; Fig. 31) and dorsally; face without tubercles; scapus, fore coxa and trochanter at most weakly compressed; epistomal suture without large depressions (Fig. 30); inner side of antennal sockets not protruding (Figs 16, 30); hypoclypeal depression large; labrum visible, smooth; clypeus straight ventrally (Fig. 30); mandible robust, gradually widened basally and with ventro-basal carina, its dorsal tooth robust (Figs 13, 31); malar suture deep and long (Figs 30, 31); pronotum short and subvertical anteriorly and pronope deep and rather large; notauli largely absent on mesoscutal disc (Figs 14, 26); mesoscutum with medio-posterior depression; scutellar sulcus narrow to medium-sized (Figs 14, 26); precoxal sulcus absent; mesopleuron with transverse carina below anterior subalar depression in type species (Fig. 22); postpectal carina absent medio-ventrally (Fig. 25); vein 1-SR of fore wing 0.3–0.5× as long as vein 1-M; vein 3-SR of fore wing 1.9–2.1× longer than vein 2-SR; hind tibia without basal carina; laterope large (Fig. 22); dorsope absent; tergites II and III smooth and of subequal length or tergite II somewhat shorter, tergite II with pair of oblique depressions basally; epipleuron of tergite III similarly sclerotised as its notum laterally and largely gently folded under notum; second metasomal suture largely absent (Figs 1, 22, 27); tergite IV clearly visible (Figs 22, 27); ovipositor sheath far protruding, its setose part 0.6–1.2× as long as fore wing; hypopygium medium-sized and acute apically (Figs 22, 28).

#### Distribution.

Palaearctic: three species.

### ﻿Key to species of the genus *Cavopius* gen. nov.

**Table:** 

1	Setose part of ovipositor sheath 0.6–0.7× as long as fore wing (Fig. 22); vein 1-M of fore wing ~ 2× as long as vein 1-SR (Fig. 11); face laterally yellowish brown	***C.daghoides* (Zaykov & Fischer, 1983)**
–	Setose part of ovipositor sheath 0.9–1.2× as long as fore wing (Fig. 1); vein 1-M of fore wing 3–4× as long as vein 1-SR (Figs 2, 24); face laterally black or dark reddish brown (Figs 7, 30)	**2**
2	Setose part of ovipositor sheath ~ 1.2× as long as fore wing (Fig. 1); metasomal tergites IV–VI broadly depressed and membranous antero-medially (Figs 1, 27, 28); vein 1-M of fore wing 3× as long as vein 1-SR (Fig. 24); antenna of ♀ with ~ 33 segments; curved setae of ventral 1/2 of occiput conspicuous (Figs 26, 31)	***C.depressorius* sp. nov.**
–	Setose part of ovipositor sheath ~ 0.9× as long as fore wing; tergites IV–VI flat and evenly sclerotised; vein 1-M of fore wing 4× as long as vein 1-SR (Fig. 2); antenna of ♀ with ~ 28 segments; curved setae of ventral 1/2 of occiput less conspicuous (Fig. 9)	***C.daghestanicus* (Telenga, 1950)**

### ﻿ Cavopius daghestanicus

Taxon classificationAnimaliaHymenopteraBraconidae

(Telenga, 1950) comb. nov.

C3C31A20-6630-53C8-B4B1-873C97833121

Figs 2–10

Opius daghestanicus Telenga, 1950: 306; Fischer 1961: 7, 1971: 60.
Opius (Misophthora) daghestanicus ; Fischer 1972: 228, 241; Papp 1981: 64.
Opius (Agnopius) daghestanicus ; Fischer 1982: 22, 1983: 15–17 (redescription).
Opius (Allotypus) daghestanicus ; Tobias and Jakimavičius 1986: 55 (transl. 79); Tobias 1998: 596.

#### Type material.

***Holotype***, ♀ (ZISP), “Dagestan, Hodshan-Maha, 28. vi.[19]26, g. Rubov”, “*Opiusdaghestanicus* sp. n., N. Telenga det.”, “Zoological Institute St. Petersburg, INS_HYM_ 0002787”.

#### Diagnosis.

**Figures 2–10.**
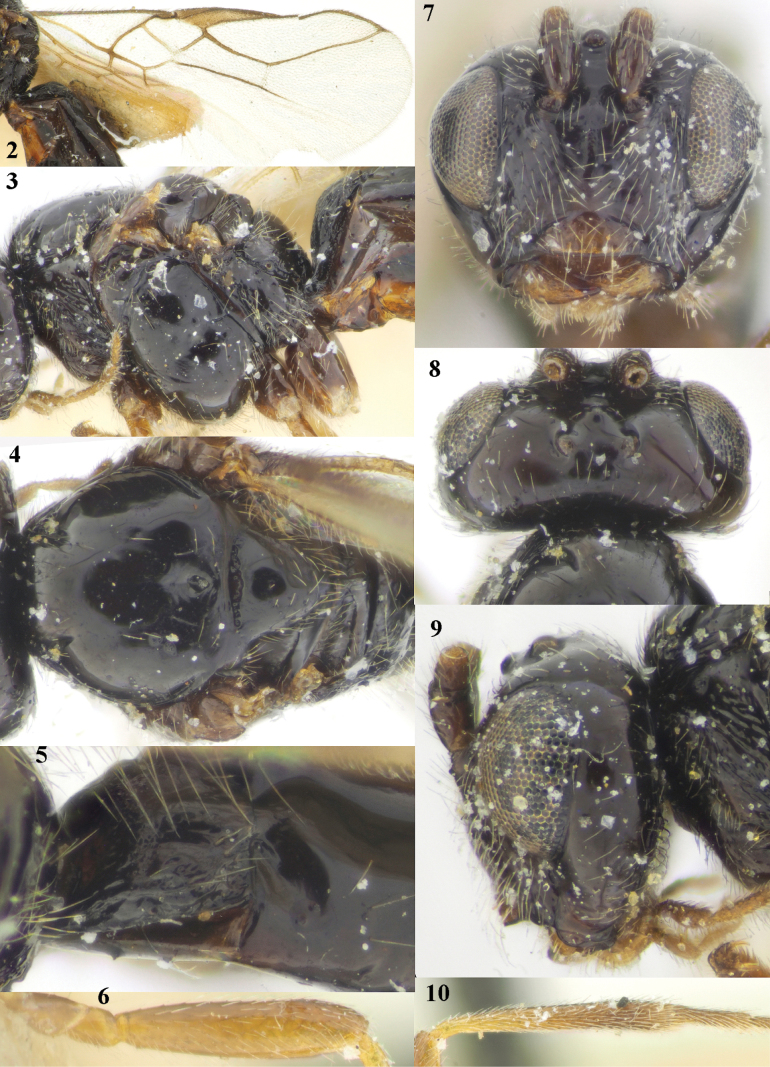
*Cavopiusdaghestanicus* (Telenga), comb. nov., holotype, ♀, Russia (Dagestan) **2** wings **3** mesosoma lateral **4** mesosoma dorsal **5** metasomal tergite I latero-dorsal **6** hind femur lateral **7** head anterior **8** head dorsal **9** head lateral **10** hind tibia lateral. Photographs by K. Samartsev.

Antenna of ♀ with approximately 28 segments; face laterally black or dark brown; ventral half of occiput less conspicuously setose (Fig. 9); vein 1-M of fore wing 4× as long as vein 1-SR (Fig. 2); metasomal tergite I rather narrowed posteriorly (Fig. 5); metasomal tergites IV–VI flat and evenly sclerotised; setose part of ovipositor sheath approx. 0.9× as long as fore wing.

#### Biology.

Unknown.

#### Distribution.

Moldova, Russia (N. Caucasus, Siberia).

#### Notes.

Photographs of the damaged holotype were very kindly supplied by Konstantin Samartsev (ZISP), showing clearly the more or less curved occipital setae (Fig. 9) which were overlooked by Fischer (1983).

### ﻿ Cavopius daghoides

Taxon classificationAnimaliaHymenopteraBraconidae

(Zaykov & Fischer, 1983) comb. nov.

6546A8C5-4EA1-5139-A3C2-86C2D79F16E1

Figs 11–23

Opius (Agnopius) daghoides Zaykov & Fischer, 1983: 41–44.
Phaedrotoma daghoides ; Jiménez-Peydró and Peris-Felipo 2011: 475.

#### Type material.

***Holotype***, ♀ (RMNH), “Bulgaria, ex coll. Zaykov, RMNH Leiden 1991”, “Rhodopi, Konush [= village near Plovdiv at foothills of Rhodope Mts, c. 190 m], 3.vi.1975, A. Zaykov”, “♀ *Opiusdaghoides* n. sp., Holotype, det. Fischer, 1982 / Holotype Fischer & Zaykov”, “Opius (Misophthora) sp. n., ♀, det. Papp J., 1981 / aff. daghestanicus Tel., 26-art. ”, “19”.

#### Diagnosis.

**Figures 11–23.**
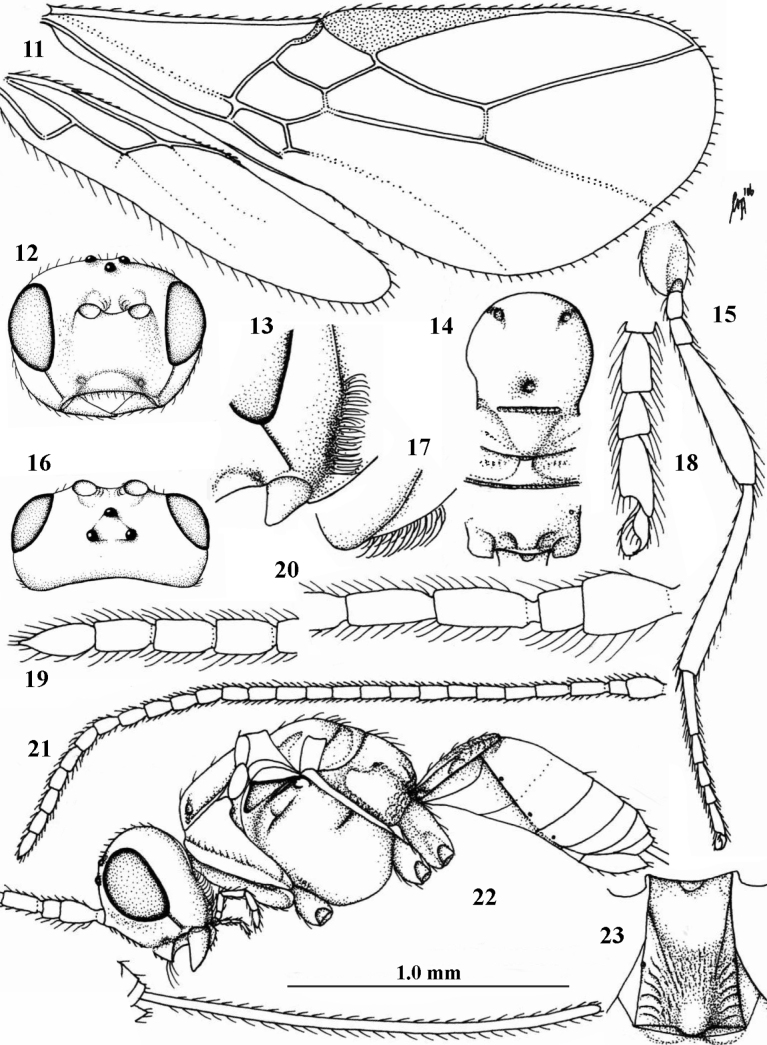
*Cavopiusdaghoides* (Zaykov & Fischer), comb. nov., holotype, ♀, Bulgaria (Konush) **11** wings **12** head anterior **13** ventral half of occiput and mandible lateral **14** mesosoma dorsal **15** hind leg **16** head dorsal **17** ventral half of occiput latero-posterior **18** outer hind tarsal claw **19** apex of antenna **20** base of antenna **21** antenna **22** habitus lateral **23** metasomal tergite I dorsal. Scale bar: 1.0× (**11, 12, 14–16, 21, 22**); 1.5× (**23**); 1.6× (**13, 17**); 2.5× (**18–20**).

Antenna with 26 segments (♀); face laterally yellowish brown; curved setae of ventral half of occiput conspicuous (Figs 13, 22); vein 1-M of fore wing ~ 2× as long as vein 1-SR (Fig. 11); setose part of ovipositor sheath 0.6–0.7× as long as fore wing (Fig. 22)

#### Biology.

Unknown.

#### Distribution.

Bulgaria, Spain.

### ﻿ Cavopius depressorius sp. nov.

Taxon classificationAnimaliaHymenopteraBraconidae

EFE62027-4A47-5237-800F-0F2DAD3247C5

https://zoobank.org/204BFF47-4F5C-4860-BCB1-3A3670348578

Figs 1 , 24–33

#### Type material.

***Holotype***, ♀ (RMNH), “S. Korea: Kangwondo, Cuncheon Nam-myon, Hudong-Li, Mal[aise] tr[ap] in half shadow at forest edge, 25.v.–14.vi.2003, P. Tripotin, RMNH”.

#### Diagnosis.

**Figures 24–33.**
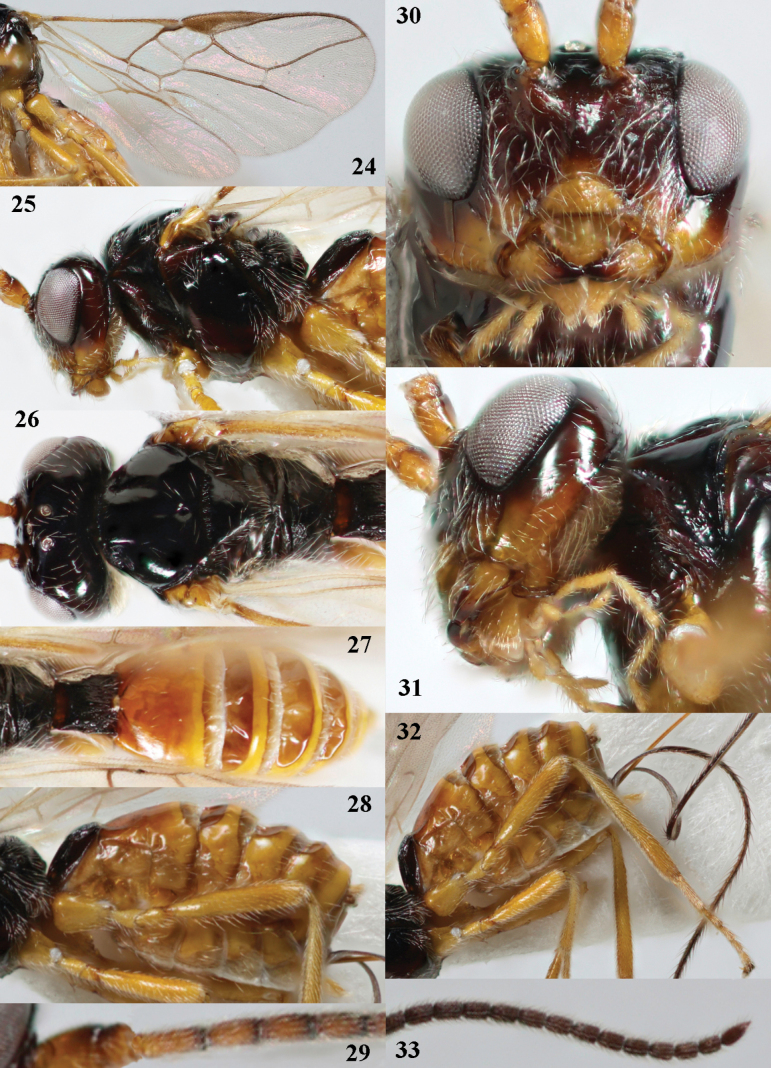
*Cavopiusdepressorius* sp. nov., holotype, ♀, S. Korea (Hudong-Li) **24** wings **25** head and mesosoma lateral **26** head and mesosoma dorsal **27** metasoma dorsal **28** metasoma lateral **29** base of antenna **30** head anterior **31** head lateral **32** hind leg lateral **33** apex of antenna.

Antenna with 37 segments (♀); curved setae of ventral half of occiput conspicuous (Figs 25, 31); face laterally black or dark reddish brown (Fig. 30); vein 1-M of fore wing 3× as long as vein 1-SR (Fig. 24); tergites IV–VI broadly depressed and membranous antero-medially (Figs 1, 27, 28); setose part of ovipositor sheath ~ 1.2× as long as fore wing (Fig. 1).

#### Description.

Holotype, ♀, length of body 3.2 mm, of fore wing 3.4 mm.

***Head*.** Antenna with 37 segments and 1.2× as long as fore wing; third segment 1.2× longer than fourth segment, length of third, fourth and penultimate segments 1.5×, 1.2× and 1.4× their width, respectively (Figs 29, 33); width of head 2.1× its median length in dorsal view, smooth dorsally and posteriorly; behind stemmaticum with indistinct depression; vertex flattened and sparsely setose; OOL: diameter of ocellus: POL= 3:1:2 (Fig. 26); anterior half of frons shallowly depressed and smooth, its posterior half smooth and laterally setose (Fig. 26); face largely smooth, shiny and with conspicuous, long setae (Fig. 30); clypeus distinctly convex, semi-circular, largely smooth (except punctulation because of very long setae) and its ventral margin thin and straight, width of clypeus 2.8× its maximum height and 0.5× minimum width of face; hypoclypeal depression large and deep (Fig. 30); eye in dorsal view 1.4× longer than temple and temple behind eye subparallel-sided (Fig. 26); occipital carina distinct but dorsally and ventrally (behind malar space) absent (Fig. 31); temple and malar space smooth; length of malar space 1.1× basal width of mandible and 0.4× height of eye; malar suture deep and complete; mandible rather twisted apically but upper tooth robust, basally symmetrical or nearly so, basal half with ventral carina (Figs 30, 31); length of maxillary palp 1.1× height of head; labial palp segments robust.

***Mesosoma*.** Length of mesosoma 1.2× its height (Fig. 25); laterally pronotum smooth but anteriorly and posteriorly with crenulate groove; dorsal pronope round and rather large; propleuron weakly and evenly convex, shiny and smooth (Fig. 31); mesopleuron sparsely punctate; postpectal carina absent; precoxal sulcus absent and its area smooth; pleural sulcus smooth; mesosternal sulcus narrow and finely crenulate; metapleuron smooth and long setose (Fig. 25); mesoscutum steeply raised above pronotum, shiny and smooth except anteriorly; notauli only impressed in anterior 1/3 of mesoscutum, rather deep and anteriorly crenulate (Fig. 26); medio-posterior depression of mesoscutum deep, round and medium-sized; transverse suture of mesoscutum present; scutellar sulcus deep and broad medially, with eight carinae and medially 0.3 × as long as scutellum (Fig. 26); scutellum largely smooth, punctulate, weakly convex, without subposterior depression; side of scutellum smooth (Fig. 26); propodeum largely smooth and lacking carinae, but medio-posteriorly somewhat elevated, rugose and antero-laterally punctate, latero-posteriorly with lamella (Figs 25, 26).

***Wings*.** Fore wing (Fig. 25): pterostigma elongate triangular, 4.5× as long as its maximum width and gradually merging into vein 1-R1; vein M+CU1 weakly curved and only sclerotised in distal quarter; vein r-m present; 1-R1 ending just before wing apex; r:2-SR:3-SR:r-m:SR1 = 5:21:42:11:72; vein r slightly widened, arising before middle of pterostigma and 2-SR sinuate; m-cu postfurcal and nearly straight, gradually merging into 2-CU1; cu-a postfurcal and vertical; 1-CU1 slightly widened; CU1b medium-sized (Fig. 24). Hind wing: M+CU:1-M:1r-m = 22:20:10; cu-a slightly curved; m-cu absent.

***Legs*.** Hind femur, tibia and basitarsus 5.3×, 8.7× and 4.8× as long as wide, respectively (Fig. 32); hind femur with dense, long setae.

***Metasoma*.** Tergite I 1.1× as long as wide apically and slightly widened apically, its surface convex medially and largely rugulose-punctate, dorsal carinae rather weakly developed and nearly up to apex of tergite (Fig. 27); tergite II and following tergites smooth; second suture absent dorsally; apex of tergites III–VI and antero-medially tergites IV–VI membranous and slightly sclerotised (more or less depressed in dead specimen; Figs 27, 28); setose part of ovipositor sheath 1.21× as long as fore wing, 8.1× tergite I and 3.6× as long as hind tibia; hypopygium acute ventro-apically and approximately as long as tergite I (Fig. 28).

***Colour*.** Black; temple chestnut brown; mandible largely, clypeus, malar space largely, palpi and legs pale yellow; tergite II and following tergites brownish yellow, but membranous parts more or less brown; antenna (but ventro-basally yellowish) dark brown; pterostigma and veins brown; ovipositor sheath dark brown; wing membrane subhyaline (Fig. 24).

#### Distribution.

Korea.

#### Biology.

Unknown.

#### Etymology.

Named after the broadly depressed metasomal tergites IV–VI (Figs 27, 28); “*depressus*” is Latin for “pressed down, low”.

### ﻿ Pseudosteres gen. nov.

Taxon classificationAnimaliaHymenopteraBraconidae

BDE3D271-9210-5365-BE7A-9D6F70EE8228

https://zoobank.org/CCA60A06-1470-46E9-BB50-0C26C5BD7ABB

Figs 34–44

#### Type species.

*Biosteresadanaensis* Fischer & Beyarslan, 2005.

#### Etymology.

From “*pseudos*” (Greek for “fallacy”) and the generic name *Biosteres* Foerster, because it is similar to *Biosteres*, but differs considerably as indicated below. Gender: masculine.

#### Diagnosis.

**Figures 34–44.**
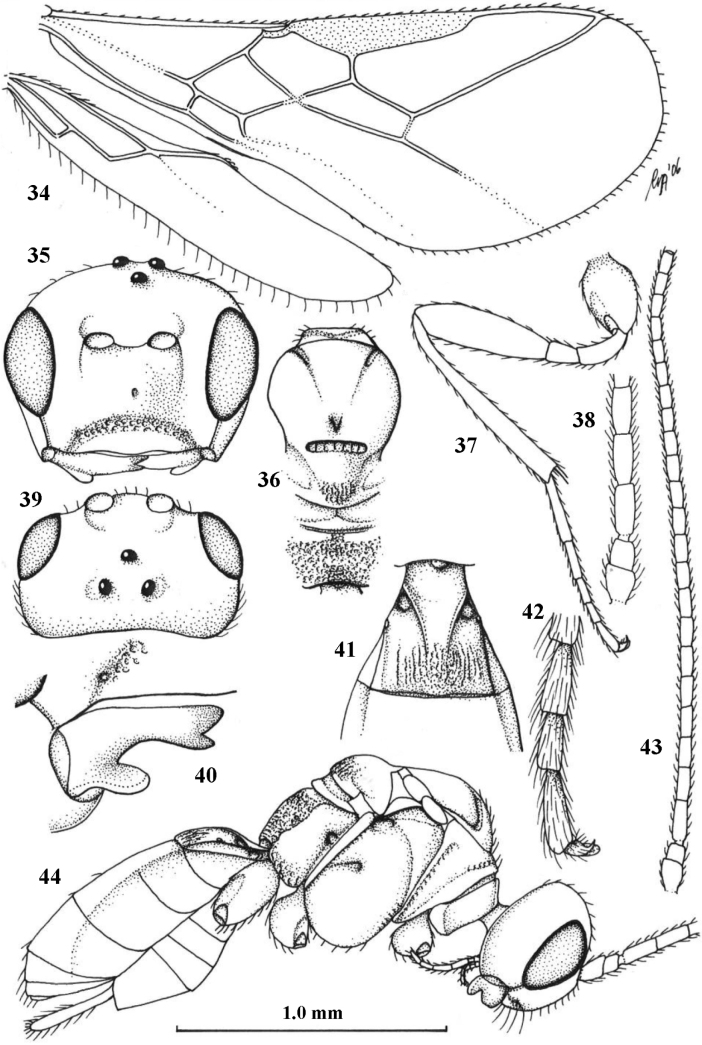
*Pseudosteresadanaensis* (Fischer & Beyarslan), comb. nov., holotype, ♀ Turkey (Adana-Balcali) **34** wings **35** head anterior **36** mesosoma dorsal **37** hind leg **38** base of antenna **39** head dorsal **40** mandible lateral **41** metasomal tergite I dorsal **42** outer hind claw lateral **43** antenna **44** habitus lateral. Scale bar: 1.0× (**34, 36, 37, 43, 44**); 1.3× (**35, 39**); 1.5× (**41**); 2.5× (**38, 40, 42**).

Hypoclypeal depression usually medium-sized, and medially ventral margin of clypeus above upper level of condyles of mandibles, but depression absent in *P.riphaeus* and narrow in *P.adanaensis* (Fig. 35); mandible with a large ventral tooth and its outer side convex (Fig. 40), mandible not twisted apically and second tooth clearly visible; notauli largely absent posteriorly (Fig. 36); medio-posterior depression of mesoscutum present; scutellum punctate medio-posteriorly; precoxal sulcus either absent, as a smooth and narrow suture or depressed and distinctly crenulate; precoxal sulcus without a second sculptured sulcus below; vein m-cu of fore wing slightly converging to vein 1-M posteriorly (Fig. 34) or parallel; vein r more or less angled with vein 3-SR of fore wing; vein 3-SR of fore wing 1.2–1.6× longer than vein 2-SR; vein m-cu of fore wing antefurcal or interstitial; pterostigma elliptical (Fig. 34) or elongate triangular; hind tibia without oblique carina basally; dorsope present (Fig. 41); hypopygium of ♀ truncate.

#### Distribution.

Palaearctic: five species.

#### Notes.

Most species are similar to the genus *Biosteres* Foerster, 1863, but differ by having a large ventro-basal tooth or lobe and in part of species also by the presence of a medium-sized hypoclypeal depression. Members of *Opiostomus* Fischer have also the mandibles basally widened and the dorsope developed, but the mandible is distinctly twisted medially, the second submarginal is much longer and the scutellum is smooth posteriorly (but punctate in *O.leptostigma* (Wesmael)).

### ﻿Key to species of the genus *Pseudosteres* gen. nov.

**Table:** 

1	Antenna of ♀ with ~ 44 segments; vein r of fore wing emitted medially from pterostigma; hypoclypeal depression absent; [vein 3-SR of fore wing ~ 1.2× vein 2-SR; precoxal sulcus distinctly sculptured; scutellum convex and finely rugulose; vein r from middle of pterostigma; mesoscutum with pair of brownish stripes; metasoma (except tergite I) reddish brown; head densely setose dorsally; notauli absent on disc; mesoscutum punctate medially; propodeum coriaceous-rugulose]; Iran, Russia	***P.riphaeus* (Tobias, 1986)**
–	Antenna of ♀ with 20–33 segments; vein r of fore wing emitted before middle from pterostigma (Fig. 34); hypoclypeal depression present, medium-sized, but narrow in *P.adanaensis* (Fig. 35)	**2**
2	Antenna of ♀ with ~ 33 segments (of ♂ with 32 (according to label of holotype, but in description 35) segments; length of body 2.5–3.0 mm and of fore wing ~ 3.0 mm; vein 1-R1 almost reaching apex of fore wing; vein m-cu of fore wing curved; [vein 3-SR of fore wing ~ 1.6× longer than vein 2-SR; ventro-basal lobe of mandible obtuse and distinctly protruding outwards; medio-posterior depression of mesoscutum large; precoxal sulcus distinctly crenulate; clypeus 5× wider than high; orbita of head largely yellow; length of hind femur (♂) ~ 3× longer than wide; vein m-cu of fore wing antefurcal; setose part of ovipositor sheath as long as metasomal tergite I]; England, Georgia, Greece, Ukraine (Crimea), and Asian part of Turkey	***P.christenseni* (Papp, 1982)**
–	Antenna of ♀ with 20–25 segments (♂ unknown); length of body 1.5–2.1 mm and of fore wing 1.7–2.3 mm; vein 1-R1 of fore wing remaining distinctly removed from apex of wing (Fig. 34); vein m-cu of fore wing straight; [pronotum, hind coxa and metasomal tergite II orange brown; setose part of ovipositor sheath 0.05–0.08× as long as fore wing and 0.5–0.7× as long as tergite I; vein r of fore wing 0.5–0.6× as long as width of pterostigma; vein CU1b of fore wing absent]	**3**
3	Clypeus slightly sinuate ventrally, largely strongly shiny and comparatively weakly widened medially (Fig. 35); vein m-cu of fore wing interstitial (Fig. 34); medio-posterior depression of mesoscutum triangular (Fig. 36); scutellum black; Asian Turkey	***P.adanaensis* (Fischer & Beyarslan, 2005)**
–	Clypeus straight ventrally, weakly shiny and distinctly widened medially; vein m-cu of fore wing antefurcal; medio-posterior depression of mesoscutum narrow elliptical; scutellum orange brown or chestnut brown	**4**
4	Vein SR1 of fore wing ~ 2.5× as long as vein 3-SR; lateral lobes of mesoscutum largely yellowish brown; [tergite I medio-posteriorly weakly striate; scutellar sulcus with distinct carinae; mesopleuron dorsally, mesoscutum, prothorax yellowish brown and metasomal tergite I black]; Ukraine	***P.arenaceus* (Jakimavičius, 1986)**
–	Vein SR1 of fore wing ~ 3.5× as long as vein 3-SR; lateral lobes of mesoscutum black; [tergite I medio-posteriorly striate; scutellar sulcus crenulate; mesopleuron largely (except dorsally) and tergite I blackish or dark brown]; Asian Turkey	***P.pseudarenaceus* (Fischer & Beyarslan, 2005)**

### ﻿ Pseudosteres adanaensis

Taxon classificationAnimaliaHymenopteraBraconidae

(Fischer & Beyarslan, 2005) comb. nov.

897D45BD-0183-5E03-9432-D879359AC1B1

Figs 34–44

Biosteres (Biosteres) adanaensis Fischer & Beyarslan, 2005: 380–382; Beyarslan and Fischer 2013: 404.

#### Type material.

***Holotype***, ♀ (NMW), “[Turkey], Adana-Balcali, 9. iv. 1985, A. Beyarslan”, “Holotype, ♀, *Biosteresadanaensis* sp. n., M. Fischer det. 2003”.

#### Diagnosis.

Antenna of ♀ with > 25 segments (♂ unknown); hypoclypeal depression narrow; clypeus slightly sinuate ventrally, largely strongly shiny and comparatively weakly widened medially (Fig. 35); vein m-cu of fore wing interstitial (Fig. 34); medio-posterior depression of mesoscutum triangular (Fig. 36); scutellum black; precoxal sulcus absent (Fig. 44); pterostigma wide elliptical (Fig. 34); vein 1-R1 of fore wing 0.6–0.7× as long as pterostigma, remaining distinctly removed from apex of wing; vein r of fore wing 0.5–0.6× as long as width of pterostigma; vein CU1b of fore wing absent; vein m-cu of fore wing interstitial (Fig. 34); vein 3-SR of fore wing 1.3× longer than vein 2-SR; pronotum, hind coxa and metasomal tergite II orange brown; setose part of ovipositor sheath 0.05–0.08× as long as fore wing and 0.5–0.7× as long as tergite I.

#### Biology.

Unknown.

#### Distribution.

Turkey (Asian part).

### ﻿ Pseudosteres arenaceus

Taxon classificationAnimaliaHymenopteraBraconidae

(Jakimavičius, 1986) comb. nov.

B8E01927-271F-5EB0-B0B4-1BE08735B897

Opius (Allotypus) arenaceus Jakimavičius (in Tobias & Jakimavičius), 1986: 63; Fischer 1991: 152–154 (redescription); Beyarslan et al. 2017: 329 [holotype, ♀ (ZISP) not examined].

#### Biology.

Unknown.

#### Distribution.

Ukraine.

#### Notes.

According to Fischer and Beyarslan (2005)*O.arenaceus* is closely related to *Opiuspseudarenaceus* Fischer & Beyarslan from Asian Turkey which belongs to *Pseudosteres* gen. nov. and, therefore, *O.arenaceus* is provisionally transferred to this genus.

### ﻿ Pseudosteres christenseni

Taxon classificationAnimaliaHymenopteraBraconidae

(Papp, 1982) comb. nov.

79F54C3B-F10B-53F3-A834-42605EEBA21A

Opius (Xynobius) christenseni Papp, 1982: 185; Fischer 1986: 618–620 (redescription); Tobias and Jakimavičius 1986: 29; Beyarslan and Fischer 2013: 447.

#### Type material.

***Holotype***, ♂ (MTMA), “Greece, Peloponnese, Monemvasia”, “15. iv. 1978, J. Papp”, “Holotypus ♂ Opius (Xynobius) christenseni sp. n., Papp, J., 1980% / ant. 32-art., “Hym. Typ. No. 2844, Museum Budapest”.

#### Biology.

Unknown.

#### Distribution.

England, Greece, Ukraine, Georgia, and Asian Turkey.

#### Notes.

As indicated on the label the holotype has 32 antennal segments, but according to the original description and the redescription the holotype male should have 35 antennal segments.

### ﻿ Pseudosteres pseudarenaceus

Taxon classificationAnimaliaHymenopteraBraconidae

(Fischer & Beyarslan, 2005) comb. nov.

1683C8BD-90AE-545B-A4D7-850E9AB0D976

Opius (Allotypus) pseudarenaceus Fischer & Beyarslan, 2005: 407–409; Beyarslan and Fischer 2013: 416.

#### Type material.

***Holotype***, ♀ (NMW), “Turkey, Adana-Balcali, 4. vi. [19]80, [A.] Beyarslan”, “Holotype, ♀, Opius (Allotypus) pseudarenaceus sp. n., det. M. Fischer, 2003”.

#### Biology.

Unknown.

#### Distribution.

Turkey (Asian part).

#### Notes.

If *Opiusarenaceus* indeed has a distinct dorsope, then *P.pseudarenaceus* and *P.arenaceus* are very similar and *P.pseudarenaceus* may be only a colour variety of the latter. The differences given by Fischer and Beyarslan (2005) are of very limited value: mesosoma 1.3× as long as high (1.4× in *P.arenaceus*), scutellar sulcus crenulate (with only three carinae), tergite I striate (rugose) and mesopleuron largely black (red).

### ﻿ Pseudosteres riphaeus

Taxon classificationAnimaliaHymenopteraBraconidae

(Tobias, 1986) comb. nov.

9AE2B4F5-56C8-54D1-B57A-A5C99B1AC3E4

Opius riphaeus Tobias, 1986: 11, 12, 23 [holotype, ♀ (ZISP) not examined].
Opius (Opiostomus) riphaeus ; Fischer, 1991: 180–182; Ameri et al. 2014: 7; Gadallah et al. 2016: 22.

#### Biology.

Unknown.

#### Distribution.

Iran, Russia (Asian part: Central Ural).

### ﻿ Cephaloplites

Taxon classificationAnimaliaHymenopteraBraconidae

Szépligeti, 1897

DB7FA4EE-099C-5A77-851E-9288CEB15FE0

Figs 45 , 46–55 , 56–67 , 68–70

Cephaloplites Szépligeti, 1897: 600; Fischer 1972: 475–477. Type species (by monotypy): Cephaloplitesmocsaryi Szépligeti, 1897.

#### Diagnosis.

**Figure 45.**
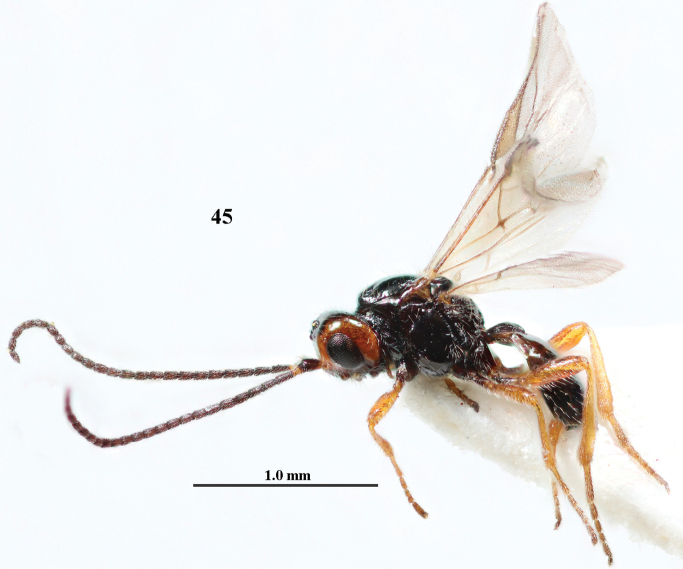
*Cephaloplitesgijswijti* sp. nov., holotype, ♂, Greece (Nemea), habitus lateral.

**Figures 46–55.**
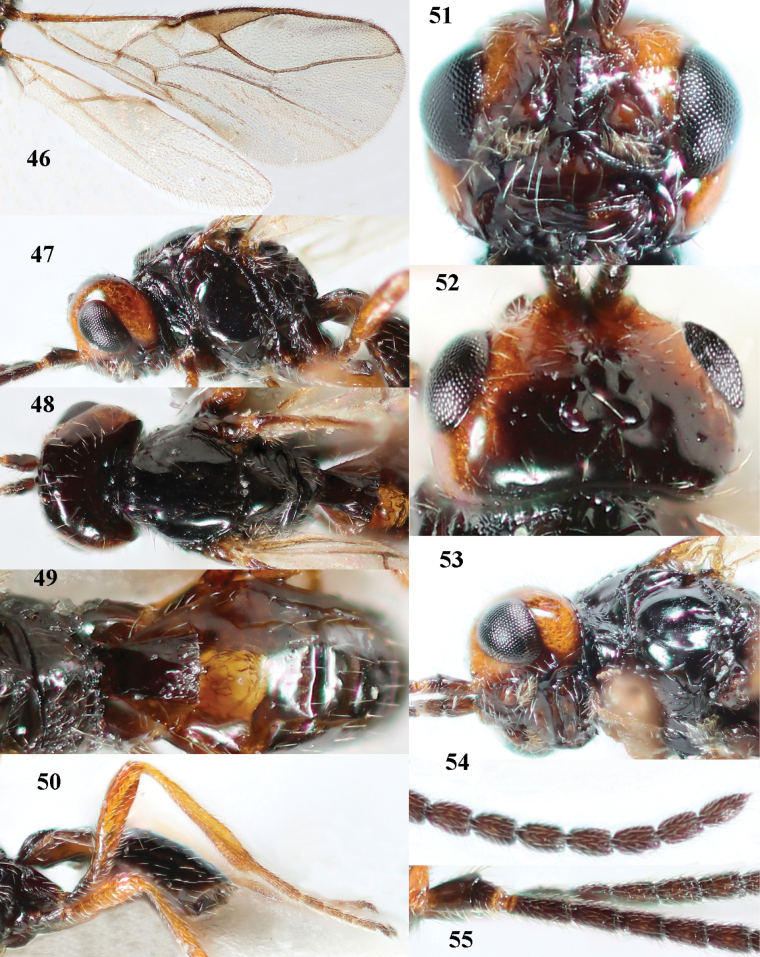
*Cephaloplitesgijswijti* sp. nov., holotype, ♂, Greece (Nemea) **46** wings **47** head and mesosoma lateral **48** head and mesosoma dorsal **49** metasoma dorsal **50** hind leg lateral **51** head anterior **52** head dorsal **53** head ventro-lateral **54** apex of antenna **55** base of antenna.

**Figures 56–67.**
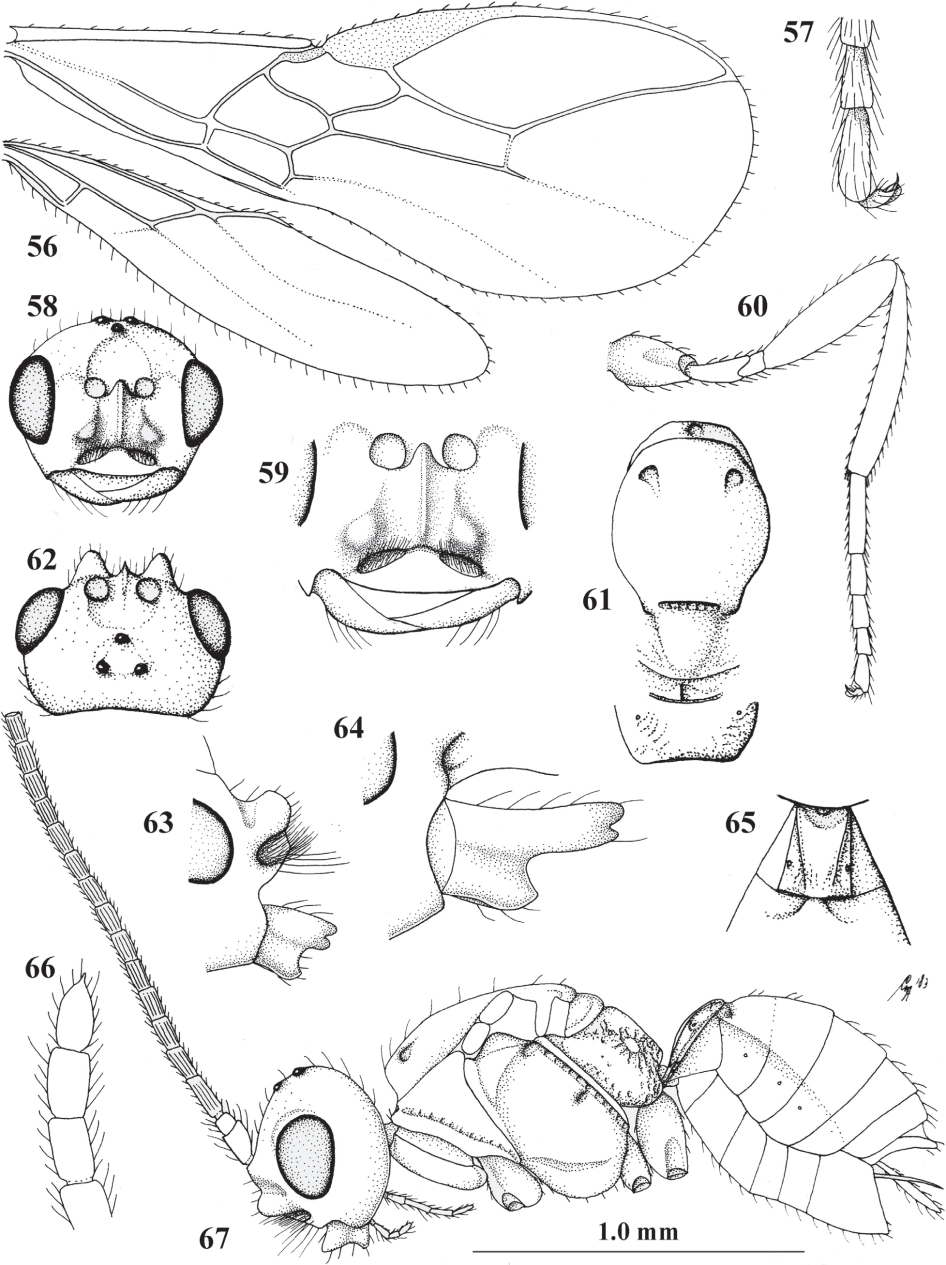
*Cephaloplitesmocsaryi* Szépligeti, ♀, Hungary (Budapest), but apex of antenna of Germany (Stuttgart) **56** wings **57** outer hind claw lateral **58** head anterior **59** face anterior **60** hind leg **61** mesosoma dorsal **62** head dorsal **63** face and mandible lateral **64** mandible lateral **65** metasomal tergite I dorsal **46** apex of antenna **67** habitus lateral. Scale bar: 1.0× (**56, 58–62, 65, 67**); 1.5× (**63, 64**); 2.5× (**57, 66**).

**Figures 68–70.**
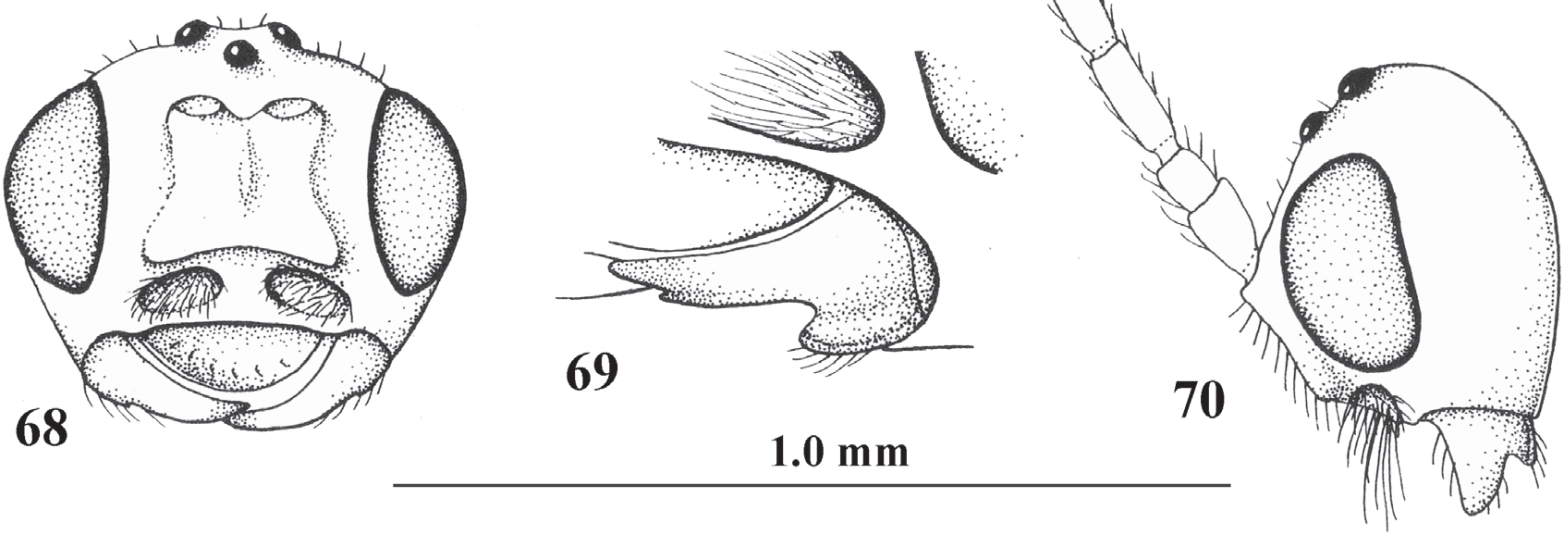
*Cephaloplitestadzhicus* Tobias & Saidov, holotype, ♂, Tajikistan **68** head anterior **69** mandible lateral **70** head lateral. Scale bar: 1.0×, but detail of mandible 1.5×.

Antenna comparatively short, approximately as long as fore wing; scapus, fore coxa and trochanter distinctly compressed; face with pair of facial tubercles below antennal sockets more or less developed (Figs 45, 51, 53, 58, 67); epistomal suture with pair of large oblique and long pubescent tentorial depressions below tubercles (Figs 51, 59, 63, 68); clypeus narrow laterally, triangular and flattened (Fig. 59); occipital carina widely absent medio-dorsally and ventrally remaining far removed from hypostomal carina; hypoclypeal depression nearly absent to medium-sized (Figs 53, 58, 68); malar suture partly present (Fig. 51) or absent (Figs 58, 67); mandible strongly widened basally (Figs 63, 64, 67, 69) and more or less twisted apically; crenulate depression above eye absent; pronope rather large and round (Fig. 61); prepectal and postpectal carinae absent (Fig. 53); notauli reduced to a pair of droplet-shaped isolated depressions (Fig. 61); medio-posterior depression of mesoscutum absent; scutellar sulcus narrow (Fig. 61); pterostigma narrowed subapically (Figs 46, 56); vein 3-SR of fore wing distinctly longer than vein 2-SR (Figs 46, 56); vein m-cu of hind wing weakly developed (Figs 46, 56); vein SR1 of fore wing completely sclerotised, reaching margin of wing and resulting in a closed marginal cell (Fig. 46); medio-posteriorly scutellum without elevated area; metasomal tergite I without dorsope; tergite II smooth and approximately as long as tergite III; ovipositor sheath hardly or not protruding beyond apex of metasoma (Fig. 67).

#### Biology.

Parasitoids of Agromyzidae (Fischer 1964).

#### Distribution.

Palaearctic: three species.

### ﻿Key to species of the genus *Cephaloplites* Szépligeti

**Table:** 

1	Head black; pair of tubercles of face indistinctly developed, only as a pair of small bumps, invisible in dorsal view of head and slightly visible in lateral view (Fig. 70); setose tentorial depression close to apical margin of clypeus and comparatively wide (Fig. 68); mandible with distinct ventral carina (Fig. 69)	***C.tadzhicus* Tobias & Saidov, 1995**
–	Head largely reddish or brownish yellow (Fig. 45); pair of tubercles of face distinctly developed, distinctly visible in dorsal view of head (Fig. 62) and in lateral view (Figs 45, 67); setose tentorial depression either more removed from apical margin of clypeus (Fig. 51) or distinctly narrower (Fig. 59); ventral carina of mandible at most slightly developed (Fig. 64)	**2**
2	Setose tentorial depression smaller and closer to base of mandible (Fig. 59); mesoscutum, scutellum and metasomal tergite III largely brownish yellow; head completely brownish yellow or largely so; coxae and trochanters brownish yellow or largely so; metasomal tergite I 1.1× as long as wide apically (Fig. 65); malar suture absent (Fig. 67)	***C.mocsaryi* Szépligeti, 1897**
–	Setose tentorial depression larger and further removed from base of mandible (Fig. 51); mesoscutum, scutellum and tergite III blackish; head partly black; coxae and trochanters dark brown or black; tergite I 1.3× longer than wide apically (Fig. 49); malar suture distinct (Fig. 51)	***C.gijswijti* sp. nov.**

### ﻿ Cephaloplites gijswijti sp. nov.

Taxon classificationAnimaliaHymenopteraBraconidae

C32218A1-C90D-53E5-A4CF-79945C5E1136

https://zoobank.org/AB164E12-6837-486D-A5B2-45DB1E2FFBDB

Figs 45 , 46–55

#### Type material.

***Holotype***, ♂ (RMNH), “Ellas [= Greece], Pelepon[nesos], prov. Korinthia, M.J. Gijswijt”, “Nemea, 20.iv.1989”.

#### Diagnosis.

Pair of tubercles of face distinctly developed, distinctly visible in dorsal view of head (Fig. 52) and in lateral view (Fig. 45); setose tentorial depression distinctly removed from apical margin of clypeus and large (Fig. 51); malar suture distinct (Fig. 51); head largely reddish or brownish yellow in lateral view (Fig. 45) and partly black in dorsal view; ventral carina of mandible at most slightly developed (Fig. 51); tergite I ~ 1.3× longer than wide apically (Fig. 49); mesoscutum, scutellum and tergite III blackish; coxae and trochanters dark brown or black.

#### Description.

Holotype, ♂, length of body 1.7 mm, of fore wing 2.0 mm.

***Head*.** Antenna with 25 segments and as long as fore wing; third segment 1.3× longer than fourth segment, length of third, fourth and penultimate segments 3.0×, 2.1× and 1.7× their width, respectively, and apical segment with minute spine (Figs 54, 55); width of head 2× its median length in dorsal view, mainly smooth dorsally and posteriorly; behind stemmaticum without distinct depression; vertex convex and sparsely setose; OOL: diameter of ocellus: POL = 7:3:4 (Fig. 52); frons shallowly depressed medially and mainly smooth, medio-posteriorly with groove (Fig. 52; eye in dorsal view 1.1× longer than temple and temple behind eye subparallel-sided (Fig. 52); face with pair of distinctly protruding convex and smooth tubercles (Figs 45, 53), visible in dorsal view of head (Fig. 52); long setose tentorial depression comparatively large and distinctly removed from base of mandible (Fig. 51); clypeus flat, triangular, smooth, shiny and its ventral margin thin and straight, width of clypeus 3.8× its maximum height and 0.8× minimum width of face; hypoclypeal depression medium-sized and deep (Fig. 51); occipital carina distinct but dorsally absent (Fig. 52); temple and malar space smooth; length of malar space 0.6× basal width of mandible and 0.2× height of eye; malar suture distinct, narrow and complete (Fig. 51); mandible weakly twisted apically, upper tooth slender, basally asymmetrical because of wide ventral lobe and no distinct ventral carina (Fig. 51); length of maxillary palp 0.7× height of head.

***Mesosoma*.** Length of mesosoma 1.3× its height (Fig. 47); laterally pronotum smooth except some crenulae and rugulae anteriorly; dorsal pronope round (Fig. 52), rather large and oblique; propleuron flattened, shiny and smooth; mesopleuron smooth and shiny; postpectal carinae absent; precoxal sulcus absent and area flat and smooth; pleural sulcus finely crenulate; mesosternal sulcus deep and moderately crenulate; metapleuron smooth but ventrally rugose (Fig. 47); mesoscutum steeply raised above pronotum, shiny and smooth anteriorly; notauli as pair of droplet-shaped and isolated depressions of mesoscutum, rather deep and anteriorly crenulate; transverse suture of mesoscutum absent; scutellar sulcus shallow and narrow, medially 0.1× as long as scutellum (Fig. 48); scutellum smooth, weakly convex, without subposterior depression; side of scutellum smooth; propodeum largely smooth, no carinae, but medio-posteriorly somewhat elevate, punctate and with some transverse striae but no lamella.

***Wings*.** Fore wing (Fig. 46): pterostigma triangular, distally elongate, 3.6× longer than its maximum width and gradually merging into vein 1-R1; vein M+CU1 weakly curved and unsclerotised; vein r-m present; 1-R1 ending just before wing apex; r:2-SR:3-SR:r-m:SR1 = 3:16:22:7:49; veins r and 3-SR slightly widened, vein r arising before middle of pterostigma and 2-SR slightly sinuate; m-cu postfurcal and straight, angled with 2-CU1; cu-a postfurcal and vertical; 1-CU1 very short and widened; CU1b rather short (Fig. 46). Hind wing: M+CU:1-M:1r-m = 7:8:5; cu-a straight; m-cu faintly indicated.

***Legs*.** Hind femur, tibia, and basitarsus 3.3×, 7.7×, and 3.0× as long as wide, respectively (Fig. 50); hind femur shiny and with long setae.

***Metasoma*.** Tergite I 1.3× longer than its apical width and slightly widened apically, its surface convex medially and largely smooth (only some rugulae posteriorly), dorsal carinae weakly developed and nearly up to apex of tergite (Fig. 49); tergite II and following tergites smooth; second suture absent dorsally.

***Colour*.** Black; temple, frons largely and face laterally reddish yellow; remainder of face, clypeus, malar space, antenna and tergite II dark brown; palpi brown; coxae and trochanters black or dark brown, remainder of legs brownish yellow; pterostigma and veins brown; wing membrane subhyaline (Fig. 46).

#### Biology.

Unknown.

#### Distribution.

Greece.

#### Etymology.

Named after the aimable collector of the holotype, Martinus Johannes (Theo) Gijswijt (10.xi.1927–27.v.2015), who was one of the major specialists of European Chalcidoidea.

### ﻿ Cephaloplites mocsaryi

Taxon classificationAnimaliaHymenopteraBraconidae

Szépligeti, 1897

A55E53A5-FB28-5B32-B599-43810048E4FD

Figs 56–67

Cephaloplites mocsaryi Szépligeti, 1897: 600–601; Fischer 1972: 476–477; Papp 2004: 157 (type lost).
Opius (Hypocynodus) kilisanus Fischer & Beyarslan, 2005: 403–405; Beyarslan and Fischer 2013: 421. Syn. nov.

#### Type material.

***Holotype*** of *C.mocsaryi* (♀ from Hungary, Budapest, Zugliget) is lost. ***Holotype*** of *O.kilisanus*, ♂ (NMW), “[Turkey], Hatay-Kilis, 6. v. 1985, A. Beyarslan”.

#### Biology.

Parasitoids of Agromyzidae: *Agromyzawoerzi* Groschke (Fischer 1964).

#### Distribution.

Czech Republic, Germany, Hungary, and Turkey (Asian part).

#### Notes.

The holotype of *O.kilisanus* has the mandible, malar space, temple ventrally, bases of hind and middle coxae and of trochantelli, mesosoma (except mesoscutum, scutellum, dorsal part of pronotum and mesopleuron) and metasoma (except second and most of tergite III) blackish, the temple somewhat less rounded and narrowed than figured for the female and the antenna with 26 segments. The differences are most likely clinal and considered to fall within the species limits of *C.mocsaryi*.

### ﻿ Cephaloplites tadzhicus

Taxon classificationAnimaliaHymenopteraBraconidae

Tobias & Saidov, 1995

0BA84853-C0D1-5FC5-825A-FC6A737E9DC5

Figs 68–70

Cephaloplites tadzhicus Tobias & Saidov, 1995: 683–684.

#### Type material.

***Holotype***, ♂ (ZISP), “[Tajikistan], Vysje, Pos. Anzov, 2000 m, Gissarsk. Chr., 26. vi. [1]965, Tobias”, “Holotypus *Cephaloplitestadzhicus* Tobias & Zaidov sp. n.”. Three male paratypes (ZISP) with same label data not examined.

#### Biology.

Unknown.

#### Distribution.

Central Asia: Tajikistan.

#### Notes.

The venation of *C.tadzhicus* is similar to that of the type species (cf. Fig. 56), the protuberances of the face are small (Fig. 70; not visible in dorsal view), the ♂ antenna with 28 segments, the metasoma dark brown as the remainder of the body, coxae, trochanters and trochantelli, the apex of the hind tibia and all tarsi infuscate to rather dark brown.

## Supplementary Material

XML Treatment for Cavopius

XML Treatment for Cavopius daghestanicus

XML Treatment for Cavopius daghoides

XML Treatment for Cavopius depressorius

XML Treatment for Pseudosteres

XML Treatment for Pseudosteres adanaensis

XML Treatment for Pseudosteres arenaceus

XML Treatment for Pseudosteres christenseni

XML Treatment for Pseudosteres pseudarenaceus

XML Treatment for Pseudosteres riphaeus

XML Treatment for Cephaloplites

XML Treatment for Cephaloplites gijswijti

XML Treatment for Cephaloplites mocsaryi

XML Treatment for Cephaloplites tadzhicus

## ﻿Additional information

### Author ORCIDs

Cornelis van Achterberg https://orcid.org/0000-0002-6495-4853

### Data availability

All of the data that support the findings of this study are available in the main text.
